# Molecular Mechanisms of TDP-43 Misfolding and Pathology in Amyotrophic Lateral Sclerosis

**DOI:** 10.3389/fnmol.2019.00025

**Published:** 2019-02-14

**Authors:** Archana Prasad, Vidhya Bharathi, Vishwanath Sivalingam, Amandeep Girdhar, Basant K. Patel

**Affiliations:** Department of Biotechnology, Indian Institute of Technology Hyderabad, Sangareddy, India

**Keywords:** amyotrophic lateral sclerosis (ALS), TDP-43, mitotoxicity, liquid-liquid phase separation (LLPS), endocytosis, frontotemporal lobar degeneration (FTLD), prion, ALS therapeutics

## Abstract

TAR DNA binding protein 43 (TDP-43) is a versatile RNA/DNA binding protein involved in RNA-related metabolism. Hyper-phosphorylated and ubiquitinated TDP-43 deposits act as inclusion bodies in the brain and spinal cord of patients with the motor neuron diseases: amyotrophic lateral sclerosis (ALS) and frontotemporal lobar degeneration (FTLD). While the majority of ALS cases (90–95%) are sporadic (sALS), among familial ALS cases 5–10% involve the inheritance of mutations in the *TARDBP* gene and the remaining (90–95%) are due to mutations in other genes such as: *C9ORF72, SOD1, FUS*, and *NEK1* etc. Strikingly however, the majority of sporadic ALS patients (up to 97%) also contain the TDP-43 protein deposited in the neuronal inclusions, which suggests of its pivotal role in the ALS pathology. Thus, unraveling the molecular mechanisms of the TDP-43 pathology seems central to the ALS therapeutics, hence, we comprehensively review the current understanding of the TDP-43's pathology in ALS. We discuss the roles of TDP-43's mutations, its cytoplasmic mis-localization and aberrant post-translational modifications in ALS. Also, we evaluate TDP-43's amyloid-like *in vitro* aggregation, its physiological vs. pathological oligomerization *in vivo*, liquid-liquid phase separation (LLPS), and potential prion-like propagation propensity of the TDP-43 inclusions. Finally, we describe the various evolving TDP-43-induced toxicity mechanisms, such as the impairment of endocytosis and mitotoxicity etc. and also discuss the emerging strategies toward TDP-43 disaggregation and ALS therapeutics.

## Introduction

TAR DNA binding protein-43 (TDP-43) was identified in 1995 as a repressor protein associated with HIV-1 transcription, which binds to the trans-active response element DNA sequence of the viral genome and is critical for the regulation of the viral gene expression (Ou et al., 1995). In 2001, TDP-43 was also reported to be involved in RNA splicing of cystic fibrosis transmembrane conductance regulator (CFTR) exons (Buratti and Baralle, 2001). It is a highly conserved and ubiquitously expressed RNA/DNA-binding protein which belongs to the large heterogeneous nuclear ribonucleoprotein (hnRNP) family, where the members of the family show ability to bind to RNA with considerable sequence-specificity achieved through the presence of one or more, highly conserved, RNA recognition motifs (RRMs) (Sephton et al., 2010, 2012; Geuens et al., 2016). TDP-43 has since then been also shown to regulate mRNAs involved in the development of neurons and embryos (Polymenidou et al., 2011; Sephton et al., 2011; Tollervey et al., 2011).

**Figure 1:**
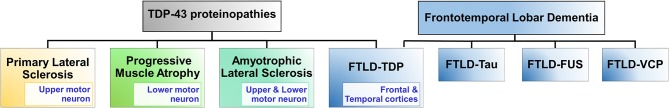
TDP-43 proteinopathies. TDP-43 proteinopathies refer to the diseases where TDP-43 is implicated and it includes: amyotrophic lateral sclerosis (ALS), frontotemporal lobar degeneration (FTLD-TDP), primary lateral sclerosis, and progressive muscular atrophy. FTLD is a group of disorders principally of the frontal temporal lobes of the brain causing dementia. Other forms of FTLD disorders are FTLD-Tau, FTLD-FUS, and FTLD-VCP. FTLD-Tau is associated with mutations in the *MAPT* gene which encodes microtubule associated protein, Tau. Tau's misfolding and aggregation lead to loss of microtubule-binding function and formation of neuronal and glial inclusions (Irwin et al., 2015). FTLD-FUS is associated with mutations in the RNA-binding protein FUS, which results in disruption of its nuclear localization and leads to its accumulation into inclusion bodies (Mackenzie et al., 2011). FTLD-VCP is associated with mutations in the valosin-containing protein (VCP). FTLD-VCP manifests ubiquitin and TDP-43-positive neuronal intranuclear and cytoplasmic inclusions. FUS, fused in sarcoma; TDP-43, TAR DNA binding protein 43; VCP, valosin containing protein.

In 2006, TDP-43 was identified as a key component of the insoluble and ubiquitinated inclusions in the brains of patients suffering from amyotrophic lateral sclerosis (ALS) and frontotemporal lobar degeneration (FTLD or FTLD-TDP) diseases (Arai et al., 2006; Neumann et al., 2006). Other diseases involving TDP-43 pathological developments are primary lateral sclerosis and progressive muscular atrophy, and together these four diseases are known as TDP-proteinopathies (Figure 1) (Dugger and Dickson, 2017). Both ALS and FTLD-TDP are late-onset neurodegenerative disorders with several common clinical, neuropathological and genetic features, however, they affect distinct regions of the nervous system (Neumann et al., 2006; Spires-Jones et al., 2017; Tan et al., 2017). Strikingly, ~97% of the ALS cases and ~45% of all FTLD cases (called: FTLD-TDP) involve TDP-43's aggregation (Ling et al., 2013; Tan et al., 2017).

ALS is a fatal neurodegenerative disease characterized by progressive degeneration of both the upper and lower motor neurons, which display cytoplasmic inclusions (Rowland and Shneider, 2001; Kiernan et al., 2011). The degradation of the upper motor neurons leads to spasticity and hyper-excitability, while the death of the lower motor neurons causes weakness, fasciculations and eventually muscular atrophy followed by progressive paralysis. The earliest symptoms include cramping and stiffness of muscles leading to muscle weakness affecting the arms and legs. The patients display slurred speech and difficulty in chewing or swallowing (Mitchell and Borasio, 2007; Rothstein, 2009). Finally, death of the patient occurs due to complications involving respiratory failure and pneumonia within about 3–5 years after the onset of disease symptoms. The average age of onset of the disease is ~50 years (Logroscino et al., 2007; Chio et al., 2009). The disease has a prevalence of ~5 individuals out of 100,000 each year worldwide. While the majority of the ALS cases (~90–95%) are considered as sporadic (sALS) with unknown cause, ~5–10% cases involve Mendelian pattern of inheritance of familial gene mutations and are known as familial ALS (fALS) (Renton et al., 2014; Taylor et al., 2016).

In addition to the TDP-43 encoding *TARDBP* gene, mutations in several other genes have also been linked with ALS such as: *SOD1* (Superoxide dismutase 1) (Rosen, 1993; Kunst et al., 1997), *FUS* (Fused in sarcoma) (Kwiatkowski et al., 2009; Vance et al., 2009), *C9ORF72* (Hexanucleotide repeat expansion in C9ORF72) (Dejesus-Hernandez et al., 2011; Renton et al., 2011), *ATXN2* (Ataxin-2) (Elden et al., 2010; Ross et al., 2011), *OPTN* (Optineurin) (Maruyama et al., 2010), *VCP* (Valosin-containing protein) (Johnson et al., 2010; Koppers et al., 2012), *PFN1* (Profilin 1) (Wu et al., 2012; Tanaka et al., 2016), *UBQLN2* and *UBQLN4* (Ubiquilin 2 and Ubiquilin 4) (Deng et al., 2011; Edens et al., 2017), *NEK1* (NIMA-like kinase 1) (Brenner et al., 2016), *MATR3* (Matrin 3) (Johnson et al., 2014b), *CHCHD10* (Coiled-coil-helix-coiled-coil-helix domain containing 10) (Woo et al., 2017), *SETX* (Senataxin) (Hirano et al., 2011), *TBK1* (TANK-binding kinase 1) (Oakes et al., 2017), and *KIF5A* (Kinesin heavy chain isoform 5A) (Nicolas et al., 2018) etc. The corresponding proteins with mutations in these genes are involved in the pathogenesis of ALS by various mechanisms.

FTLD is a progressive neuronal disease associated with the degeneration of the frontal and temporal lobes with neuronal intranuclear and cytoplasmic inclusions (Mackenzie et al., 2007; Dugger and Dickson, 2017). Unlike ALS, which rarely involves dementia, FTLD is the second most prevalent cause of dementia after the Alzheimer's disease, in individuals <65 years of age, with an estimated prevalence of ~15–22 per 100,000 (Van Langenhove et al., 2012; Onyike and Diehl-Schmid, 2013). It is characterized by significant personality and behavioral changes, as well as gradual impairment of the language skills. Strikingly, TDP-43 inclusions in FTLD-TDP are also hyper-phosphorylated, ubiquitinated and N-terminally truncated as observed in ALS (Neumann et al., 2007a; Hasegawa et al., 2008; Igaz et al., 2008). Also, mutations in the *TARDBP* gene can lead to ALS as well as the FTLD-TDP disease.

## Structure of TDP-43

**Figure 2:**
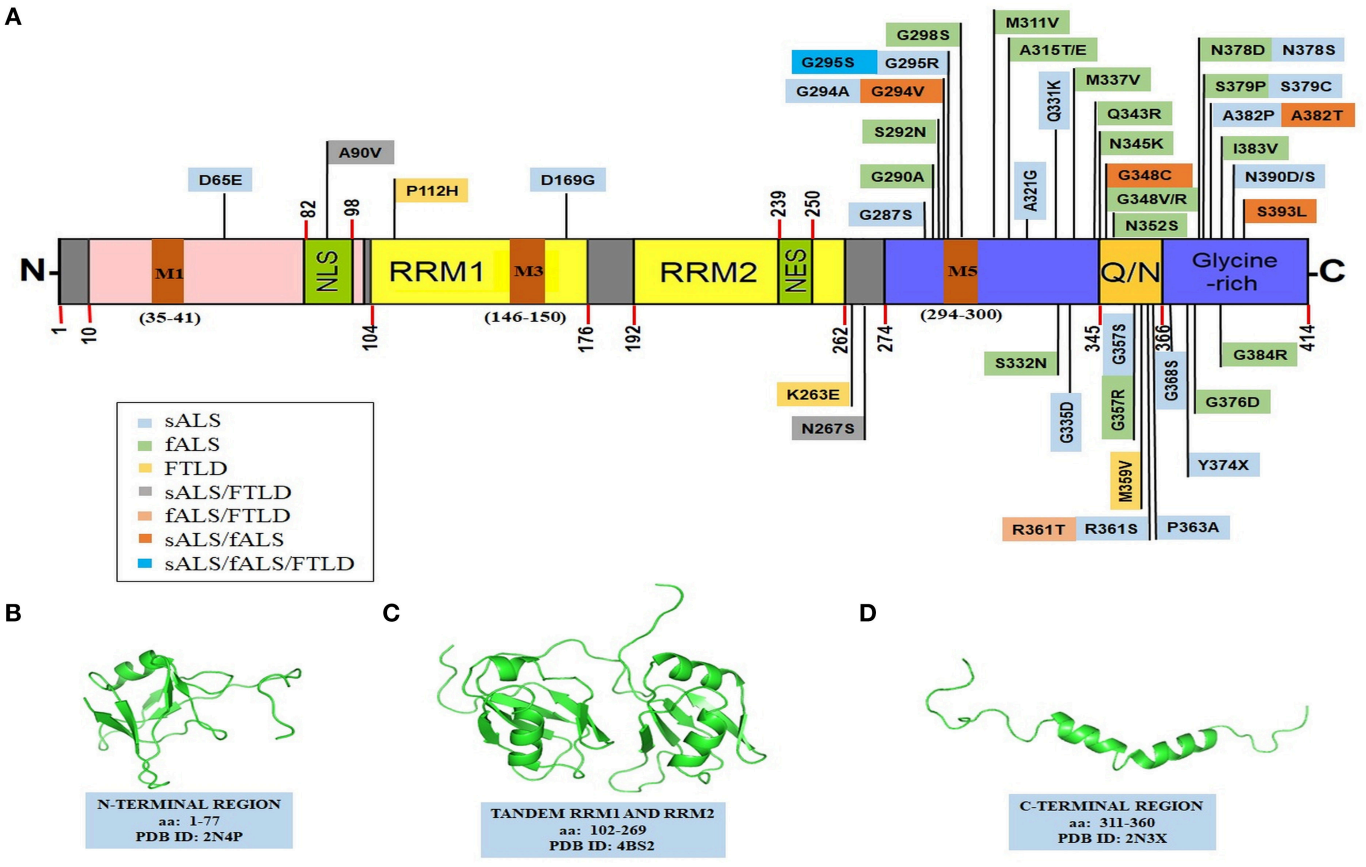
Structural features of TDP-43. **(A)** TDP-43's domain organization depicting ALS and FTLD-linked mutations. TDP-43 comprises of an NTD domain, two RRM domains, a nuclear export signal (NES), a nuclear localization signal (NLS), a prion-like disordered C-terminal domain (with glutamine/asparagine-rich (Q/N) and Glycine-rich regions) and mitochondrial localization motifs (M1−35–41; M3−146–150; M5−294–300). Sporadic mutations and familial gene mutations generating amino acid substitutions in TDP-43 are classified. Several TDP-43 mutations overlap between ALS and FTLD as well as between sALS and fALS (Baumer et al., 2009; Xiong et al., 2010; Fujita et al., 2011; Janssens et al., 2011; Budini et al., 2012; Chiang et al., 2012; Cruts et al., 2012; Lattante et al., 2013; Moreno et al., 2015). PDB structures of: **(B)** N-terminal region (PDB id-2N4P); **(C)** a tandem RRM1 and RRM2 segment (PDB id-4BS2); **(D)** a C-terminal region (aa: 311–360) (PDB id-2N3X). Structures in the **(B–D)**, have been adapted with permissions respectively from: John Wiley and Sons (Mompeán et al., 2016b); Springer Nature (Lukavsky et al., 2013); and Springer Nature (Jiang et al., 2016, creative commons attribution 4.0 license). fALS, familial amyotrophic lateral sclerosis; NES, nuclear export signal; NLS, nuclear localization signal; NTD, N-terminal domain; Q/N, glutamine/asparagine; RRM, RNA recognition motif; sALS, sporadic amyotrophic lateral sclerosis; TDP-43, TAR DNA binding protein 43.

The TDP-43 protein contains 414 amino acids and the encoding gene *TARDBP* is located on the chromosome number 1. It comprises of an N-terminal region (aa 1–102) with a nuclear localization signal (NLS, aa 82–98), two RNA recognition motifs: RRM1 (aa 104–176) and RRM2 (aa 192–262), a nuclear export signal (NES, aa 239–250), a C-terminal region (aa 274–414) which encompasses a prion-like glutamine/asparagine-rich (Q/N) domain (aa 345–366) and a glycine-rich region (aa 366–414) (Figure 2) (Cohen et al., 2011; Lukavsky et al., 2013; Kuo et al., 2014; Qin et al., 2014; Jiang et al., 2016; Mompeán et al., 2016b). TDP-43 is predominantly localized in the nucleus but also shuttles to the cytoplasm for some of its functions (Ayala et al., 2008). In ALS, there is an increase in the cytoplasmic TDP-43 concentration leading to cytoplasmic inclusion formation (Neumann et al., 2006; Winton et al., 2008a). Mitochondrial localization of TDP-43 depends on internal motifs M1 (aa 35–41), M3 (aa 146–150), and M5 (aa 294–300), which consists of continuous stretch of hydrophobic amino acids (Wang et al., 2016). Owing to its poor *in vitro* solubility and high aggregation propensity, the complete structure of TDP-43 has remained elusive thus far. Several groups, however, have determined high resolution structures of some of its domains (Figure 2) and its holistic structure is now evolving.

### N-Terminal Domain (NTD)

Accumulating evidence suggests that TDP-43 is natively dimeric or at least exists in a monomer-dimer equilibrium under normal physiological conditions (Shiina et al., 2010; Zhang Y. J. et al., 2013). TDP-43's dimerization apparently occurs through interactions of the N-terminal residues and while several reports suggest that TDP-43 N-terminal domain's (NTD) dimerization is necessary for its physiological functions like RNA splicing. Others have also argued that the NTD's dimerization may, in fact, be involved in its aggregation (Shiina et al., 2010; Zhang Y. J. et al., 2013; Afroz et al., 2017). Notably, TDP-43's N-terminal region exhibits an ubiquitin-like fold, which consists of one α-helix and six β-strands in the β1-β2-α1-β3-β4-β5-β6 arrangement (Figure 2) (Qin et al., 2014; Mompeán et al., 2016b). The homodimerization of TDP-43 molecules occurs by head-to-head interaction of the two NTDs while the RRM2 domains are extended outwards (Wang Y. T. et al., 2013). In fact, Zhang et al. have reported that the first ten residues of the NTD are crucial for the formation of the functional homodimers and are also involved in the aggregation of the full-length TDP-43 (Zhang Y. J. et al., 2013). Of note, the N-terminal region can promote self-oligomerization in a concentration-dependent manner, which modulates its nucleic acid binding properties (Chang et al., 2012). Recently, using single-molecule fluorescence technique, evidence has also been provided that the NTD undergoes reversible oligomerization, which enhances the propensity of the intrinsically disordered C-terminal region to aggregate (Tsoi et al., 2017).

In contrast, it has also been argued that the TDP-43's dimerization *via* NTD allows for the interactions with the partner proteins and the target RNAs, thereby possibly preventing its aggregation. Indeed, the dimeric TDP-43 NTD has been shown to enhance its pre-mRNA splicing activity, improve solubility and protect against the formation of the cytoplasmic TDP-43 inclusions (Jiang et al., 2017). Recently, a 2.1 Å resolution structure of the 1–80 residues of the TDP-43 NTD has revealed the presence of dynamic solenoid-like structure which spatially separates the aggregation-prone C-terminal region and probably reduces the pathological aggregation (Afroz et al., 2017). Deletion or mutation in the nuclear localization signal (NLS) sequence in the NTD induces cytoplasmic relocalization and aggregation of TDP-43 (Winton et al., 2008a; Barmada et al., 2010). In fact, the ALS-associated A90V mutation present in the nuclear localization signal (NLS) can sequester the endogenous TDP-43 into insoluble cytoplasmic aggregates (Winton et al., 2008b).

### RNA Recognition Motifs (RRMs)

RNA binding proteins (RBPs) contain highly conserved RNA recognition motifs (RRMs), which are among the most abundant protein domains in the eukaryotes (Romano and Buratti, 2013; Gerstberger et al., 2014; Marchese et al., 2016; Conlon and Manley, 2017). These proteins are involved in several RNA metabolic processes like mRNA processing, RNA export and RNA stability. Some RBPs, such as TDP-43, are also implicated in neurodegenerative diseases which therefore hints of disturbances in the RNA metabolism as a causative factor (Maris et al., 2005; Lunde et al., 2007; Clery et al., 2008). TDP-43 contains two RRM domains (RRM1 and RRM2) that are separated by 15 amino acids (Kuo et al., 2009, 2014; Lukavsky et al., 2013). These RRM domains comprise of five β-strands and two α-helices arranged in the β1-α1-β2-β3-α2-β4-β5 pattern (Lukavsky et al., 2013; Sun and Chakrabartty, 2017). Both of the TDP-43 RRM domains are involved in binding with cognate RNA/DNA molecules with higher specificity toward short UG/TG-rich sequences of the RNA/DNA molecules (Lukavsky et al., 2013; Kuo et al., 2014). Several mutations in the RRMs are shown to disrupt the RNA binding capability while not significantly interfering with the RNA recognition (Lukavsky et al., 2013). Notably, two ALS-linked missense mutations have also been identified in this region: the P112H and the caspase cleavage susceptible, D169G (Buratti, 2015; Moreno et al., 2015; Chiang et al., 2016). Proposedly, the RRM2 domain may also contribute to the dimerization of the TDP-43 protein (Kuo et al., 2009). Binding to single-stranded DNA (ssDNA) or single-stranded RNA (ssRNA), and not to double-stranded DNA (dsDNA), has been shown to enhance the TDP-43's solubility and expectedly also prevent its aggregation (Huang et al., 2013; Sun and Chakrabartty, 2017). Importantly, TDP-43 actively binds to the 3′ untranslated regions (UTRs) of several thousand mRNA transcripts, and even to its own mRNA as an autoregulation mechanism to control its own cellular concentration and possibly also its solubility (Ayala et al., 2011).

### C-Terminal Domain (CTD)

The C-terminal region of TDP-43 (aa 277–414) is highly disordered and comprises of a glycine-rich region and also a segment enriched in uncharged polar amino acids, glutamine and asparagine (Q/N) (Figure 2). This unusual composition resembles the prion-like domains of several yeast proteins, such as Sup35, Rnq1, and Cyc8 etc. (Patel et al., 2009; King et al., 2012; Liebman and Chernoff, 2012). The prionogenic domain-containing yeast proteins can switch from a disordered conformation to a self-templating, cross-β sheet-rich amyloid-like conformation, sometimes as an adaptive physiological response (Liebman and Chernoff, 2012). Strikingly, out of nearly 240 human proteins that harbor a potential prion-like domain, about 70 of them are RNA/DNA-binding proteins containing an RRM motif, several of which, including TDP-43, FUS, hnRNPs, TATA-box binding protein associated factor 15 (TAF15), and EWS RNA binding protein 1 (EWRS1) etc., are being implicated in the pathogenesis of various neurodegenerative diseases (March et al., 2016; Harrison and Shorter, 2017). The C-terminal region of TDP-43 seems of special relevance to the pathological behavior of TDP-43. Firstly, alike prion-like domains, it is intrinsically disordered and aggregation-prone (Santamaria et al., 2017). Secondly, it harbors most of the ALS-associated *TARDBP* mutations and phosphorylation sites. Thirdly, certain C-terminal fragments of sizes ~25–35 kDa produced from TDP-43 through aberrant activity of caspases, are highly cytotoxic and are the prominent species found in the inclusion bodies identified from the ALS-affected brains (Zhang et al., 2007, 2009). The C-terminal region of TDP-43 also contains a short, highly dynamic and unstable helix-turn-helix region (aa 311–360) (Jiang et al., 2013, 2016). Peptides from this region can efficiently form amyloid-like fibrils *in vitro*, which can exhibit prion-like infectious seeding ability to cells expressing the soluble TDP-43 (Chen et al., 2010; Guo et al., 2011; Jiang et al., 2013). Interestingly, TDP-43 C-terminal region can also undergo liquid-liquid phase separation (LLPS) to form dynamic protein droplets. Within these droplets, the C-terminal residues show mild transient interactions, that appear crucial for stress granule formation (Conicella et al., 2016). Mutations, persistent stress conditions, or aging, are proposed to cause these droplets to undergo a liquid-to-solid phase separation (LSPS), thereby forming irreversible pathological aggregates (Patel et al., 2015).

## Physiological Functions of TDP-43

### TDP-43-RNA Interactions

**Figure 3:**
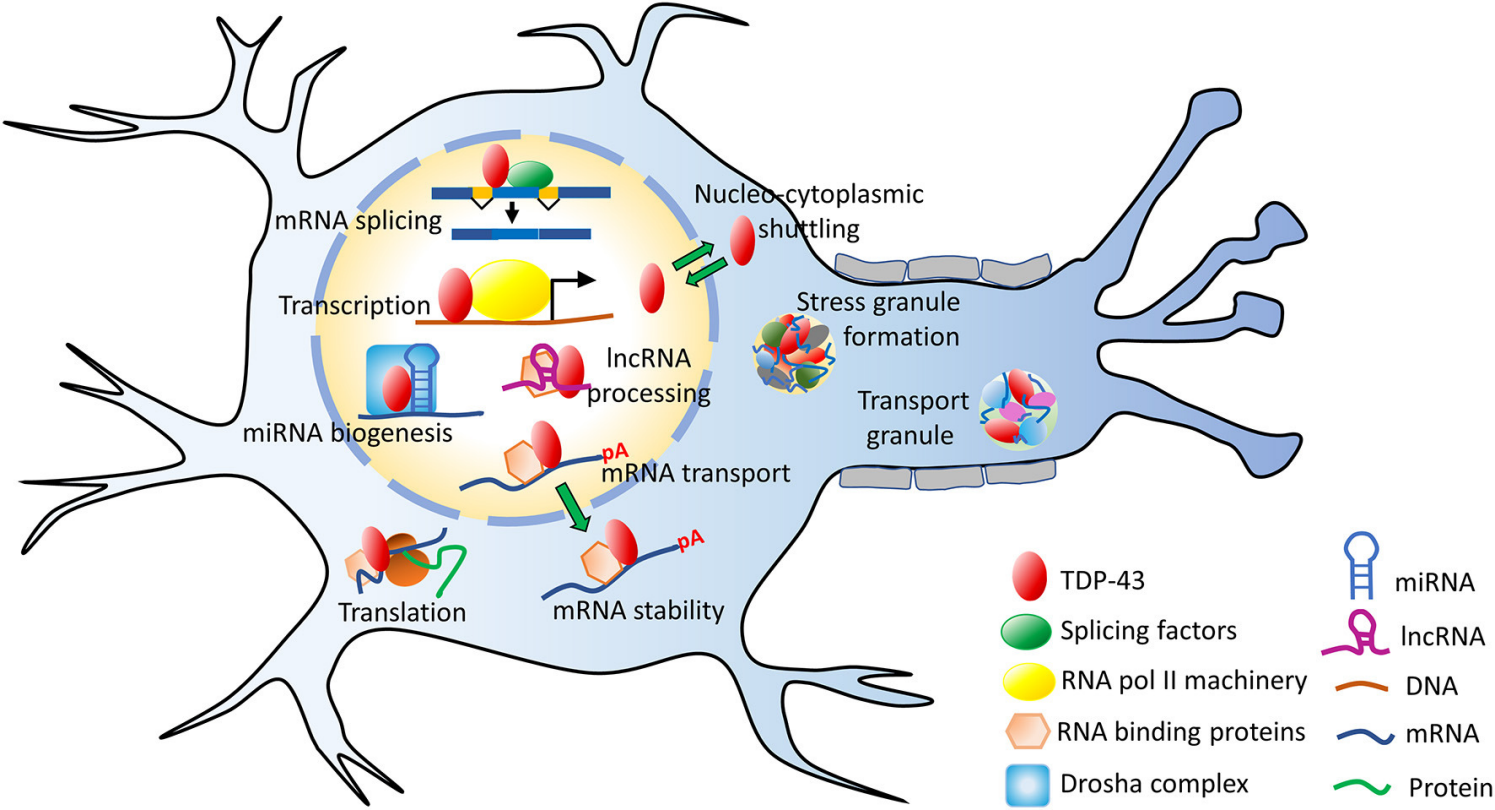
Functions of TDP-43. TDP-43 performs several mRNA-related processes in the nucleus, such as transcription, splicing, maintaining RNA stability as well as miRNA and lncRNA processing. It is predominantly a nuclear protein but also shuttles between the nucleus and the cytoplasm. In the cytoplasm, TDP-43 participates in the stress granule formation, ribonucleoprotein (RNP) transport granule formation, translation and other processes. lncRNA, long non-coding RNA; miRNA, microRNA; mRNA, messenger RNA; pA, poly-A mRNA tail; TDP-43, TAR DNA binding protein 43.

TDP-43 has versatile functions and it is involved in several steps of RNA metabolism such as: transcription, translation, mRNA transport, mRNA stabilization, microRNA (miRNA) and long non-coding RNA (lncRNA) processing etc. (Ling et al., 2013; Coyne et al., 2017) (Figure 3). Using genome-wide RNA immunoprecipitation techniques (CLIP-seq), more than 6,000 mRNA targets were identified to associate with TDP-43, which would be nearly 30% of the entire transcriptome (Polymenidou et al., 2011; Tollervey et al., 2011; Xiao et al., 2011). Earlier conventional RNA immunoprecipitation methods, have also revealed specific RNA targets (Buratti and Baralle, 2001; Sephton et al., 2011). While TDP-43 binds with high specificity to the UG-rich sequences of RNAs, it mostly binds to the 3′ UTRs of mRNAs/pre-mRNAs when localized to the cytoplasm (Colombrita et al., 2012). This suggests a broad role of TDP-43 in maintaining mRNA stability, maturation and transport (Tollervey et al., 2011; Colombrita et al., 2012).

#### mRNA Transcription and Splicing

TDP-43 is absent from the areas of silent heterochromatin but localizes to the sites of transcription and splicing (Casafont et al., 2009). It regulates the splicing patterns of transcripts of several important genes, such as Cystic fibrosis transmembrane conductance regulator (*CFTR), TARDBP, FUS, SNCA* (α-synuclein), *HTT* (Huntingtin), and *APP* (Amyloid precursor protein) etc. (Buratti and Baralle, 2001; Polymenidou et al., 2011, 2012). In fact, nuclear depletion of TDP-43 results in mRNA splicing aberrations (Arnold et al., 2013; Highley et al., 2014; Yang et al., 2014). Likewise, over-abundance of TDP-43 could form dysfunctional complexes, due to limited supply of the binding partner proteins. Indeed, imbalances caused by the overexpression of TDP-43 are detrimental to the neuronal cells (Cannon et al., 2012; Heyburn and Moussa, 2016; Lu et al., 2016). The nuclear depletion of TDP-43 was also found to trigger widespread dysregulation of the splicing events in the motor neurons (Highley et al., 2014). Two ALS-associated mutations in TDP-43, Q331K, and M337V, have also been shown to alter mRNA splicing processes in a transgenic mice model (Polymenidou et al., 2011, 2012; Lagier-Tourenne et al., 2012; Arnold et al., 2013).

#### mRNA Maturation and Stability

By binding with mRNA transcripts, TDP-43 regulates stabilities of several mRNAs, including that of its own mRNA (Strong et al., 2007; Volkening et al., 2009; Ayala et al., 2011; Colombrita et al., 2012; Costessi et al., 2014). TDP-43 interacts with regulatory 3′ UTR sequences of these mRNAs and affects their half-life, either positively, as observed for the human low molecular weight neurofilament mRNA, or negatively, as documented for the vascular endothelial growth factor and progranulin mRNA transcripts (Strong et al., 2007; Volkening et al., 2009; Ayala et al., 2011; Colombrita et al., 2012; Costessi et al., 2014).

#### mRNA Transport

TDP-43 associates with the RNA molecules to produce ribonucleoprotein (RNP) granules which transport mRNA to distant locations. In the axonal cells, RNP granules are trafficked with assistance from microtubules (Alami et al., 2014). In fact, ALS-associated TDP-43 mutants were found to impair the transportation of the RNP granules (Wang et al., 2008; Alami et al., 2014).

#### mRNA Translation

Proteomics has revealed the TDP-43's global protein interaction profile which has also identified several partner proteins involved in the RNA metabolism, such as splicing and translation. Several of these interactions were unperturbed by the ALS-linked mutations, A315T and M337V (Freibaum et al., 2010; Kim et al., 2010). Recent studies in *Drosophila*, have reported that TDP-43 regulates localization and translation of the *Futsch* (ortholog of *Map1b*) mRNA at the neuromuscular junctions (Coyne et al., 2014). TDP-43 can also form complexes with other proteins involved in the translation machinery, for example: the ribosomal protein, receptor for activated C kinase 1 (RACK1) (Russo et al., 2017). In one study, an increase in cytoplasmic TDP-43 caused repression of the global protein synthesis in the neuroblastoma cells, which could be rescued by the over-expression of RACK1 (Russo et al., 2017). TDP-43 can also alter the translation of several mRNAs *via* sequestration of the translation factors into stress granules (Aulas and Vande Velde, 2015).

#### Stress Granule Formation

Eukaryotic cells have developed several mechanisms that protect cells against diverse cellular insults. The formation of stress granules (SG), the membrane-less cytoplasmic foci of sizes ≤5 μm, ensues quickly upon exposure to stresses like: oxidative stress, heat shock, viral infection, and chemical exposure etc. (Anderson and Kedersha, 2009; Aulas and Vande Velde, 2015). SGs are usually safe “storage and sorting stations” for RNA binding proteins, translationally stalled mRNAs and arrested pre-initiation complexes. The formation of SG is a reversible process and SGs dissolve after the stress is over (Anderson and Kedersha, 2008). Neuronal cells are quite vulnerable to stress, and a defective stress response may facilitate the conversion of SGs into pathological inclusion bodies as seen in the ALS and FTLD-affected brains (Wojcik et al., 2006; Van Damme et al., 2008; Colombrita et al., 2009; Dormann and Haass, 2011). TDP-43 is capable of assembling into stress granules, indicating its protective role against cellular insults (Colombrita et al., 2009; Aulas and Vande Velde, 2015). In fact, TDP-43 is involved in both assembly and maintenance of SGs, and it also regulates the expression of key SG nucleating proteins, rasGAP SH3 domain binding protein 1 (G3BP) and T cell-restricted intracellular antigen-1 (TIA-1) (McDonald et al., 2011). ALS-linked mutations can influence stress granule dynamics. Under sorbitol-induced osmotic stress, the G348C mutant TDP-43 was found to be localized into progressively larger stress granules (Dewey et al., 2011). On the contrary, the R361S mutant of TDP-43 was shown to disrupt the stress granule assembly (McDonald et al., 2011). The abnormal effects of several other ALS-associated mutations on stress granule dynamics is discussed furthermore in the “role of TDP-43 mutations” section of this review.

#### miRNA and lncRNAs Processing

TDP-43 also promotes biogenesis and processing of the non-coding RNAs, such as microRNA (miRNA) (Kawahara and Mieda-Sato, 2012). Recent studies have confirmed of the interactions of TDP-43 with the Drosha and Dicer complexes (Ling et al., 2010; Kawahara and Mieda-Sato, 2012). TDP-43 associates with the nuclear Drosha complex and binds directly to the primary miRNAs to facilitate the production of a subset of precursor miRNAs (pre-miRNAs) (Kawahara and Mieda-Sato, 2012). In human embryonic kidney 293 cells (HEK293), it is found that the cytoplasmic TDP-43 interacts with the Dicer complex and promotes pre-miRNA processing (Kawahara and Mieda-Sato, 2012). In fact, TDP-43's down-regulation leads to altered expression of several miRNAs in the cultured HeLa cells, rodent neurons and induced pluripotent stem cells (iPSC)-derived human neurons (Buratti et al., 2010; Zhang Z. et al., 2013). In genome-wide studies, several long non-coding RNAs (lncRNAs), which are transcripts of >200 nucleotides, that do not encode for proteins but regulate gene expression through various mechanisms, such as nuclear enriched abundant transcript 1 (NEAT1) and metastasis associated in lung adenocarcinoma transcript 1 (MALAT1), were found to bind with TDP-43. Interestingly, NEAT1 and MALAT1 are also found at elevated levels in FTLD-TDP (Tollervey et al., 2011).

### TDP-43 Protein-Protein Interactions

A global interactome study has revealed that TDP-43 interacts with proteins involved in diverse physiological functions (Freibaum et al., 2010). In a recent study, Blokhuis et al. have performed an interactome analysis to identify binding partners of ALS-associated proteins in neuronal cells using immunoprecipitation, pull down assays and mass spectrometry. Many DNA- and RNA-binding proteins were detected in the interactome of TDP-43 which are involved in RNA processing, gene expression, RNA splicing, post-transcriptional regulation of gene expression and translation (Blokhuis et al., 2016). TDP-43 has either direct physical interactions, or RNA-dependent interactions, with several proteins and some of the key interactions have been outlined in the Table 1.

**Table 1:** Key interactions of TDP-43 protein with other proteins.

**Protein**	**Remarks**	**Reference(s)**
**RNA-BINDING PROTEINS**		
FUS	TDP-43 interacts with a small fraction of FUS. ALS mutations in TDP-43 enhance interaction with FUS. Perturbation of this interaction was observed to reduce the expression of histone deacetylase 6 (HDAC6) mRNA.	Kim et al., 2010; Ling et al., 2010; Kabashi et al., 2011
hnRNPA1 and hnRNPA2/B1	hnRNPs interact with TDP-43 C-terminal region and regulate mRNA splicing and TDP-43's feedback auto-regulation.	Buratti et al., 2005; D'ambrogio et al., 2009; Romano et al., 2014; Blokhuis et al., 2016
TIA1	TIA1 is involved in stress granule (SG) formation and participates in direct physical or RNA-dependent association with TDP-43 in SGs. TIA1 mutations identified in ALS increase its phase separation propensity, disrupt the normal disassembly of SGs and promote the accumulation of non-dynamic SGs containing the TDP-43 protein.	Liu-Yesucevitz et al., 2010; McDonald et al., 2011; Mackenzie et al., 2017
RBM45	RBM45 accumulates in inclusion bodies in ALS and FTLD patients. RBM45 co-localizes with TDP-43's cytoplasmic aggregates. No RBM45 mutations in ALS have been reported yet. Mutations in RBM45 show propensity to form cytoplasmic aggregates which recruit TDP-43, and impair mitochondrial functions.	Collins et al., 2012; Li Y. et al., 2015; Mashiko et al., 2016
Ataxin-2	Poly-glutamine expansion in Ataxin-2 are genetic risk factor for ALS. Ataxin-2 with 22 glutamines is normal, while 27–33Qs impart ALS risk and if present with >34Qs, it is involved in spinocerebellar ataxia type 2 (SCA2). Ataxin-2 and TDP-43 physically interact in an RNA-dependent manner. Poly-glutamine expansions in ataxin-2 that have been identified in ALS enhance its stability and increase the TDP-43's cleavage and phosphorylation.	Elden et al., 2010; Ross et al., 2011; Hart and Gitler, 2012; Nihei et al., 2012; Kim et al., 2014
Matrin3	Co-immunoprecipitation experiments have revealed that Matrin3 and TDP-43 interact in an RNA-dependent manner. Matrin3's S85C mutation enhances its interaction with TDP-43.	Johnson et al., 2014b; Gallego-Iradi et al., 2015; Boehringer et al., 2017
**IMMUNE RESPONSE**		
p62 and p65 (NFκB)	TDP-43 interacts with NFκB and acts as a co-activator of NFκB in ALS patient's glial and neuronal cells inducing the production of pro-inflammatory cytokines and neurotoxic mediators.	Swarup et al., 2011
**HEAT SHOCK RESPONSE AND PROTEOSTASIS**		
Hsp40 and Hsp70	Hsp40/Hsp70 co-chaperone/chaperone system interact with TDP-43's C-terminal region and suppress heat-shock-induced TDP-43 aggregation. Heat-shock protein DNAJB2 associates with Hsp70 and regulates TDP-43's clearance by maintaining it in soluble state. Overexpression of the yeast Hsp40 homolog Sis1 reduces TDP-43 toxicity in the yeast model.	Udan-Johns et al., 2014; Chen H. J. et al., 2016; Park et al., 2017
DNAJB1 and DNAJB6	Overexpression of DNAJB1 (Hsp40 protein, mammalian Sis1 homolog) was found to reduce TDP-43-mediated toxicity in primary cortical neurons of rodent. Overexpression of DNAJB6 suppresses the formation of heat-shock-induced TDP-43 nuclear aggregates. DNAJB6 interacts with the disordered C-terminal domain of TDP-43 and modulates TDP-43 aggregation and also influences its interaction with other RNA binding partners.	Udan-Johns et al., 2014; Park et al., 2017
PDI	The chaperone PDI interacts with mutant TDP-43 and co-localizes in the spinal cord neuronal cells. PDI might also be involved in preventing the abnormal cysteine cross-linking of TDP-43.	Walker et al., 2013
Parkin	The E3-ubiquitin ligase Parkin ubiquitinates TDP-43 and forms a multi-protein complex with HDAC6 and induces sequestration of TDP-43 into cytosolic inclusions.	Hebron et al., 2013; Wenqiang et al., 2014
Ubiquilin1 and Ubiquilin2	Mutations in ubiquilin proteins are involved in aberrations in the proteasomal and autophagy pathways. Ubiquilin2 binds with high affinity to TDP-43 and induces accumulation of poly-ubiquitinated inclusions in the neuronal cells.	Kim et al., 2009; Hanson et al., 2010; Cassel and Reitz, 2013; Osaka et al., 2016
Optineurin	Optineurin mutations cause blindness and glaucoma. Recently, optineurin was found to associate with TDP-43 in ALS and sporadic inclusion body myositis.	Yamashita et al., 2013; Li C. et al., 2015
**OXIDATIVE STRESS RESPONSE**		
SOD1	ALS-linked SOD1 mutants interact with TDP-43 into detergent-insoluble fractions. Mutant SOD1 and TDP-43 co-operatively modulate the neurofilament mRNA stability.	Volkening et al., 2009; Higashi et al., 2010
CHCHD10	CHCHD10 is a mitochondrial protein found at the cristae junction in the intermembrane spaces that regulates mitochondrial structure and oxidative phosphorylation. TDP-43 interacts with CHCHD10 and induces its nuclear localization while the CHCHD10 dysfunction promotes the TDP-43's cytoplasmic accumulation.	Johnson et al., 2014a; Woo et al., 2017

*CHCHD10, coiled coil helix coiled coil helix domain containing protein 10; DNAJB1, DnaJ homolog subfamily B member 1; DNAJB6, DnaJ homolog subfamily B member 6; FUS, fused in sarcoma; HDAC6, histone deacetylase 6; hnRNP A1 and A2/B, heterogeneous nuclear ribonucleoprotein A1 and A2/B; Hsp, heat shock protein; NFκB, nuclear factor kappa-light-chain-enhancer of activated B cells; PDI, protein disulfide isomerase; RBM45, RNA-binding motif protein 45; SCA2, spinocerebellar ataxia type 2; SOD1, superoxide dismutase 1; TIA1, T cell-restricted intracellular antigen-1*.

## TDP-43 Pathology in ALS

The pathological hallmarks of TDP-43 proteinopathies include nucleus to cytoplasmic mislocalization, deposition of ubiquitinated and hyper-phosphorylated TDP-43 into inclusion bodies, protein truncation leading to formation of toxic C-terminal TDP-43 fragments, and protein aggregation. Sporadic or familial mutations can aggravate these detrimental effects and cause early disease-onset. In this section, we review these disease mechanisms in detail.

### Role of TDP-43 Mutations

Numerous mutations in the *TARDBP* gene have been identified to be associated with ALS and FTLD (Sreedharan et al., 2008; Buratti, 2015) (Figure 2). The effects of these mutations on the TDP-43 protein include: increased propensity to aggregate, enhanced cytoplasmic mislocalization, altered protein stability, resistance to proteases or modified binding interactions with other proteins etc. The role of TDP-43 mutations have also been comprehensively reviewed earlier elsewhere (Pesiridis et al., 2009; Lattante et al., 2013; Buratti, 2015). Dedicated online databases are also available that provide detailed information about geographical prevalence of these mutations (Pinto et al., 2011; Cruts et al., 2012; Abel et al., 2013). Most of the ALS-associated mutations appear in the exon 6 of the *TARDBP* gene which encodes for the C-terminal glycine-rich region of TDP-43. The most commonly occurring missense mutations are A382T and M337V and some of the most well-studied mutations are A315T, Q331K, M337V, D169G, G294A/V, and Q343R etc., for which several ALS-disease models have also been established (Buratti, 2015). TDP-43 mutations including A90V and N267S are observed in both cases of sporadic ALS as well as FTLD whereas R361T was reported in a patient case of fALS and FTLD. Mutations, such as G294V, G348C, A328T, and S393L are found in both the sporadic as well as familial cases of ALS. Interestingly, TDP-43 mutation G295S encompasses various pathological conditions including sALS, fALS, and FTLD (Baumer et al., 2009; Xiong et al., 2010; Fujita et al., 2011; Janssens et al., 2011; Budini et al., 2012; Chiang et al., 2012; Cruts et al., 2012; Lattante et al., 2013; Moreno et al., 2015). Of interest, a fALS associated phosphorylation-prone TDP-43 mutant, which contains G298S mutation in the mitochondrial localizing internal motif M5, was found to have increased import into the mitochondria (Wang et al., 2016).

Mutations in the TDP-43's C-terminal region enhance its intrinsic aggregation propensity (Johnson et al., 2009). Recombinantly expressed TDP-43 protein harboring the ALS-linked mutations, such as Q331K, M337V, Q343R, N345K, R361S, and N390D, were found to have increased aggregation *in vitro* and also promoted cytotoxicity in the yeast cells (Johnson et al., 2009). Peptides from the TDP-43's putative amyloidogenic core region (aa 286–366) containing the ALS-associated mutations were also found to efficiently form amyloid-like fibrils (Chen et al., 2010; Guo et al., 2011; Sun et al., 2011; Zhu et al., 2014) (Table 2). Interestingly, Zhu et al. have reported that an aggregated TDP-43 peptide with the A315E mutation is capable even of cross-seeding the aggregation of the amyloid-β 1–40 peptide (Zhu et al., 2014). Also, Guo et al. have shown that TDP-43 A315T forms amyloid fibrils *in vitro* and causes neuronal death when added to the cultured neuronal cells (Guo et al., 2011). Certain mutations in TDP-43 like G294V, A315T, M337V, A382T, and G376D, are also found to enhance the cytoplasmic mislocalization of TDP-43 (Barmada et al., 2010; Mutihac et al., 2015; Mitsuzawa et al., 2018).

**Table 2:** Observations on amyloid-like aggregation and oligomerization of TDP-43 and its peptides.

**TDP-43 protein or its peptides**	**Tools used**	**Observation(s)**	**Reference(s)**
**AMYLOID-LIKE AGGREGATION**			
FL TDP-43 (1–414)	ThT, CR, TEM	Amyloid-like fibrils.	Johnson et al., 2009
FL TDP-43, FL TDP-43 M337V, FL TDP-43 A382T, 193–414, 193–414 M337V, 193–414 A382T	ThT, TEM	Amyloid-like fibrils. Formation of thin fibrils that stack together to form thick bundles. Moderately bind to the ThT dye.	Furukawa et al., 2011
287–322, 287–322 A315T, 287–322 G294A, 287–322 G294V, 287–322 G294P, 287–322 G295S, 292–322, 297–322, 302–322, 307–322	ThT, TEM, CD, FTIR	Amyloid-like fibrils. Peptides 287–322 (wt and A315T) form β-sheet-rich ThT-negative fibrils. Mutants G294A, G294V, and G295S form ThT-staining fibrils. Further delineation of the amyloidogenic 287–322 fragment, shows amyloidogenic propensity of smaller peptides. Peptide 307–322 exerts disruption of membrane integrity. All peptides impart neurotoxicity.	Chen et al., 2010; Liu et al., 2013; Sun et al., 2014
286–331, 286–331 A315T	ThT, TEM, AFM	Amyloid-like fibrils. Both these 46 amino acid peptides form ThT-positive amyloid fibrils which are toxic to the neuronal cells. The A315T mutation enhances fibril formation and toxicity.	Guo et al., 2011
103–183, 103–183 C173S, 103–183 C175S	ThT, AFM	Amyloid-like fibrils. The RRM1 domain forms large globular particles and fibrillar aggregates. Cysteine to serine substitution enhances amyloidogenicity.	Shodai et al., 2013
208–265	ThT, TEM, CD, SAXS	Truncated RRM2 domain forms ThT-negative fibrillar aggregates.	Wang Y. T. et al., 2013
307–319, 307–319 A315T, 307–319 A315E	ThT, AFM, CD, FTIR	Amyloid-like fibrils. Mutant TDP-43 peptides are capable of cross-seeding the aggregation of the Alzheimer's amyloid-β peptide.	Zhu et al., 2014
246–258, 311–323, and smaller peptides from these regions.	ThT, TEM, SLS	Amyloid-like fibrils. ThT-positive amyloid aggregates. Regions, 246–255 (EDLIIKGISV) and 311–320 (MNFGAFSINP) are important sequence determinants of the TDP-43 C-terminal aggregation. DLII and NFGAF are the shortest amyloidogenic sequences.	Saini and Chauhan, 2011, 2014
318–343, 311–360, 311–360 Q331K, 311–360 G335D, 311–360 M337V	ThT, CD, AFM	Amyloid-like fibrils. Amyloidogenic core region 311–360 forms ThT-positive amyloid fibrils. G335D mutation enhances aggregation and inclusion body formation. G335D forms a loop linker between the two α-helices in this region and promotes α-to-β transition.	Jiang et al., 2013, 2016
341–357	ThT, CR, TEM, CD, XRD	Amyloid-like fibrils. Putative amyloidogenic core 341–357 peptide forms amyloid fibrils ~15–25 nm wide and several hundred nanometers long with marked tendency to associate laterally.	Mompean et al., 2015
193–414	ThT, AFM, CD	Amyloid-like fibrils. ThT-positive fibrillar aggregates having varying lengths (~1 to 2 μm) with an average height of ~20–25 nm. β-sheet content is found to be ~45%.	Furukawa et al., 2011; Prasad et al., 2016, 2018
234–273, 274–313, 314–353	ThS, TEM	Amyloid-like fibrils. 234–273 peptide formed short fibrillar aggregates. The peptides 274–313 and 314–353 formed longer, straight, or twisted fibrils (10–15 nm diameter) and displayed prion-like behavior.	Shimonaka et al., 2016
102–269	ThT, TEM, DLS	Amyloid-like fibrils. Zinc binds to RRM 1 and 2 regions and results in aggregation into rope-like aggregates.	Garnier et al., 2017
TDP-43 RRM2 region:247–252, 247–255, 247–256, 247–257, 248–253, 248–256, 248–257, 250–259, 252–257, 252–259, 253–259	XRD, MicroED, Cryo-EM	The RRM peptide 247-DLIIKGISVHI-257 forms an array of amyloid polymorphs, which fit into different classes of steric zippers and adopt different backbone conformations.	Guenther et al., 2018b
TDP-43 LCD region: 300–306, 321–326, 328–333, 333–343, 370–375, 396–402	XRD, MicroED, TEM	These segments form amyloid steric zipper structures.	Guenther et al., 2018a
TDP-43 LCD region: 312–317, 312–317 A315E, 312–317 A315T	XRD, MicroED, TEM	These segments form kinked beta-sheet structure and are involved in hydrogels and protein droplet formations alike to as observed in the membrane less organelles.	Guenther et al., 2018a
**OLIGOMERIZATION**			
FL TDP-43	TEM, AFM, DLS, Immuno-labeling	Oligomers. Spherical oligomers with particle sizes of ~40–60 nm. Oligomers are conformationally and functionally distinct from the native TDP-43 and are neurotoxic.	Fang et al., 2014
FL TDP-43 (Tandem dimer) (Amino acid residues 1–414 x2)	Immuno-blotting	Dimers. Dimeric TDP-43 induces accumulation of high molecular weight TDP-43 aggregates.	Shiina et al., 2010
FL TDP-43, TDP-43 NTD	Chemical cross-linking, TEM, NMR spectroscopy	Oligomers. N-terminal domain (NTD)-mediated TDP-43's oligomerization into solenoid-like structures. Resistant to cellular stress (cysteine-independent).	Afroz et al., 2017
NLS-TDP-25, TDP-25	FRET, FRAP, FCS, Super-resolution Fluorescence Microscopy	Oligomers. NLS-TDP-25 oligomers are ordered and non-toxic whereas TDP-25 oligomers are disordered and toxic. NLS-TDP-25 does not incorporate into cytoplasmic inclusion bodies.	Kitamura et al., 2017
FL TDP-43	Immuno-labeling, TEM	Oligomers. TDP-43 forms neurotoxic spherical oligomers in the human brain. TDP-43 oligomers are abundant in the FTLD-TDP type C brain.	Kao et al., 2015

*AFM, atomic force microscopy; CD, circular dichroism; CR, Congo red; Cryo-EM, cryo-electron microscopy; FCS, fluorescence correlation spectroscopy; FL, full-length; FRAP, fluorescence recovery after photobleaching; FRET, fluorescence resonance energy transfer; FTIR, fourier-transform infrared spectroscopy; LCD, low complexity domain; MicroED, micro-electron diffraction; NLS, nuclear localization signal; NMR, nuclear magnetic resonance spectroscopy; NTD, N-terminal domain; SAXS, small-angle X-ray scattering; SLS, static light scattering; TEM, transmission electron microscopy; ThS, thioflavin-S; ThT, thioflavin-T; XRD, X-ray diffraction. The tools used for the analyses of the amyloid-like aggregates/aggregation have been briefly described in Box 1*.

Box 1 **Tools for the analysis of amyloid-like aggregates.**

**AFM** (Atomic Force Microscopy): AFM provides the surface contour by scanning using a molecular size cantilever and it provides surface topology of amyloid aggregates or fibers. AFM images can provide height features of amyloid fibrils/aggregates.

**CD** (Circular Dichroism Spectroscopy): By measuring the differential absorbance of circularly polarized light, CD is widely used to characterize the protein's secondary structural elements. Amyloid-like aggregates tend to show higher β-sheet structure in comparison with the soluble, monomeric protein molecules and exhibits a negative peak around 215 nm.

**CR** (Congo Red Birefringence): Upon binding with amyloid aggregates, absorbance maximum of CR shifts from 490 to 540 nm. Macroscopic amyloid aggregates bound to CR display apple-green birefringence when observed under cross-polarized light.

**Cryo-EM** (Cryo-Electron Microscopy): An electron microscopic technique used for imaging frozen-hydrated specimens at cryogenic temperatures, where the specimens remain in their native state without the need for dyes or fixatives, allowing structure determination at high resolution. Cryo-EM generated micrographs have been used to distinguish various structural classes of amyloids.

**DLS** (Dynamic Light Scattering): Fluctuation of intensity of scattered light with time due to Brownian motion of particles in solution are analyzed to detect diffusion of the molecules. DLS provides hydrodynamic radii of particles and can be used to evaluate the presence of amyloid aggregates and estimate their sizes.

**FCS** (Fluorescence Correlation Spectroscopy): FCS records fluctuations in fluorescence intensity, providing information, such as diffusion coefficient and hydrodynamic radius which are used as a measure of size and concentration of monomers and aggregates in a solution.

**FRAP** (Fluorescence Recovery After Photobleaching): A spectroscopic technique which is used to measure the diffusion of a population of fluorescently labeled molecules after photobleaching. It gives valuable insights into the mobility of intracellular aggregated species.

**FRET** (Fluorescence Resonance Energy Transfer): FRET measures energy transfer from a donor fluorophore to acceptor fluorophore and can be used to detect the presence of small sub-population of oligomeric assemblies of misfolded proteins.

**FTIR** (Fourier Transform Infrared Spectroscopy): Composition of secondary structural elements are determined by FTIR by measuring molecular bond vibrational frequencies. FTIR spectra can provide structural features of protein misfolding intermediates where the larger and rigid amyloids absorb near 1,620 cm^−1^ whereas the small and disordered fibers absorb at ~1,635 cm^−1^.

**MicroED** (Micro- Electron Diffraction): A new method of cryo-EM where diffraction patterns are collected from submicron-thick 3D crystals using a focused low-dose electron beam under cryogenic temperatures and are deployed to visualize amyloid crystals with dimensions of few hundred nanometers.

**NMR spectroscopy** (Nuclear Magnetic Resonance Spectroscopy): NMR is a spectroscopic technique to determine the molecular structure, dynamics and chemical environment of molecules by measuring magnetic fields of certain atomic nuclei. Since amyloids exhibit favorable nuclear spin relaxation, NMR is used in characterization of the overall symmetry of cross–β structures.

**SAXS** (Small-Angle X-ray Scattering): SAXS is used to determine the average particle size, shape, distribution, and surface-to-volume ratio by analyzing the elastic scattering of X-rays at small angles when passed through a specimen. This technique is widely used to characterize structural variations in amyloid fibrils.

**SLS** (Static Light Scattering): SLS uses time-averaged intensity of scattered light to estimate molecular weight of particles in a solution and thereby helps in identifying the presence of higher molecular weight amyloid-like aggregates.

**Super-resolution Fluorescence Microscopy**: In super-resolution microscopy, temporal or spatial modulation of the excitation or activation light helps to overcome the resolution limit to extract higher resolution information of the samples and provides detailed information on species morphology of oligomeric and fibrillary structures.

**TEM** (Transmission Electron Microscopy): TEM provides morphological visualization of amyloid aggregates or fibers. First, the amyloid samples are negatively stained using metal compounds, such as uranyl acetate, before imaging.

**ThS** (Thioflavin-S Fluorescence): Binding of ThS with amyloid aggregates displays a sharp fluorescence emission peak at ~520 nm when excited at 440 nm. It is also used to stain amyloid aggregates present in tissue sections and cell culture.

**ThT** (Thioflavin-T Fluorescence): Binding of the planar dye Thioflavin-T to amyloid-like aggregates increases its fluorescence emission intensity at ~485 nm when excited at 445 nm.

**XRD** (X-Ray Diffraction): Subjecting amyloid fibers to X-ray results in the display of a specific diffraction pattern known as the cross-β pattern in which β-strands run perpendicular to the fiber axis and β-sheets extend parallel to the fiber axis.

TDP-43 protein is intricately associated with stress granule dynamics (Liu-Yesucevitz et al., 2010; Walker et al., 2013). Quantification of the TDP-43 levels accumulated in the stress granules, has revealed that the ALS-linked D169G and R361S mutants accumulate in larger quantities than the wild-type TDP-43 (McDonald et al., 2011). Additionally, TDP-43 with the G348C mutation forms significantly larger stress granules, and is incorporated into the stress granules earlier than the wild-type TDP-43, although eventually, the wild-type TDP-43 expressing cells form more number of stress granules per cell, albeit, the granule sizes remain unchanged (Dewey et al., 2011). Additionally, the aggregation-enhancing A315T and Q343R mutations have been shown to increase TDP-43-containing RNA granule's average sizes, decrease their distribution density and also hamper their mobility in the neuronal cells (Liu-Yesucevitz et al., 2014). The mutations, D169G, G294A, Q343R, N390D, Q331K, and M337V, were found to enhance the formation of TDP-43-positive inclusion bodies in the neuronal cell line, SH-SY5Y (Nonaka et al., 2009a).

A plausible pathological mechanism is the alteration of the TDP-43 protein's stability by the mutations. In one study, the ALS-linked TDP-43 with the mutations G298S, Q331K, and M337V, showed longer half-life and higher stability than the wild-type TDP-43 (half-life: ~24–48 h vs. 12 h for the wild-type TDP-43) in an isogenic cell line (Ling et al., 2010). Further evidence from the works of Watanabe et al. (2013) and Austin et al. (2014), has shown that the accelerated disease onset in the familial ALS patients is related to the TDP-43 mutations (such as: A315T, Q343R, N352S, M337V, G298S, G348C, A382T, D169G, and K263E) possibly *via* increase in the protein half-lives and the aggregation propensities, which may further influence their own mRNA's processing and cause misregulation of the TDP-43's translation (Watanabe et al., 2013; Austin et al., 2014).

Certain mutations also confer increased susceptibility of TDP-43 to protease-mediated degradation (Nonaka et al., 2009b). Calpain-I could fragment the recombinant TDP-43 A315T and M337V mutant proteins more rapidly than the wild-type TDP-43, whereas the D169G mutant TDP-43 was more efficiently cleaved by caspase-3 *in vitro* (Yamashita et al., 2012; Chiang et al., 2016). Interestingly, another mutation A90V in TDP-43 imparts partial resistance to the digestion by caspase-3 (Wobst et al., 2017).

### Nuclear Depletion and Cytoplasmic Accumulation of TDP-43

One of the prominent features of ALS and FTLD-TDP, is the loss of functional TDP-43 in the nucleus and its increased deposition into cytoplasmic inclusion bodies in the brain and spinal cord neurons (Arai et al., 2006; Neumann et al., 2006). While TDP-43 is predominantly nuclear, it also shuttles between the nucleus and the cytoplasm thereby engaging in diverse functions (Ayala et al., 2008). In fact, TDP-43 interacts with several proteins involved in the mRNA splicing and other RNA metabolisms in the nucleus, and also interacts with several cytoplasmic proteins, such as those involved in the mRNA translation (Freibaum et al., 2010; Ling et al., 2013). TDP-43's cellular concentration is therefore tightly auto-regulated to maintain its steady levels *via* a negative-feedback mechanism (Ayala et al., 2011). The precise sequence of events abetting the pathological TDP-43 mislocalization is debated, however, nuclear TDP-43 depletion appears to precede the inclusion body formation (Lee et al., 2011; Xu, 2012). Notably however, it is argued that the TDP-43-associated disturbances in the mRNA metabolism may be more central, as compared to the cytoplasmic accumulation and aggregation of TDP-43, toward the pathogenesis of ALS and FTLD-TDP. It is accepted that the cytoplasmic accumulation and the aggregation of TDP-43 into inclusion bodies confer both a loss-of-function as well as a gain-of-toxic-function (Vanden Broeck et al., 2015; Ederle and Dormann, 2017). Numerous studies have supported the detrimental effects of the TDP-43's cytoplasmic aggregation in the neuronal cells (Igaz et al., 2009; Pesiridis et al., 2011; Yang et al., 2011; Wang Y. T. et al., 2013). The cytoplasmic accumulation into inclusion bodies reduces the amount of TDP-43 necessary for mRNA transport. Recently, TDP-43 has also been found to function as a translational repressor by interacting with the ribosomal protein, receptor of activated protein C kinase 1 (RACK1), thereby resulting in the global protein synthesis inhibition (Russo et al., 2017). Interestingly, RACK1 was also found to be sequestered into the TDP-43 inclusions in the motor neurons of the ALS patients (Russo et al., 2017). Cytoplasmic TDP-43 is also proposedly involved in the mitochondrial impairment, which is further discussed here in a later section (Wang et al., 2016).

The TDP-43's signal sequences, nuclear localization signal (NLS) and nuclear export signal (NES), regulate the nucleocytoplasmic shuttle of TDP-43 (Winton et al., 2008a). Deletion of the nuclear localization signal (NLS) [or nuclear export signal (NES)] sequence impairs TDP-43's functions. Expectedly, TDP-43 with the nuclear localization signal (NLS) deletion accumulates as cytoplasmic aggregates and can even sequester the native TDP-43 thereby further depleting the TDP-43 pool, which consequently alters the transcripts that regulate chromatin assembly and histone processing (Winton et al., 2008a; Amlie-Wolf et al., 2015). Likewise, a TDP-43 mutant with nuclear export signal (NES) deletion manifested a propensity to form nuclear aggregates (Winton et al., 2008a). Only one familial ALS-linked mutation in the nuclear localization signal (NLS) has been identified till date i.e., A90V, although several C-terminal mutations can also drive increased cytoplasmic localization, however, how this ensues remains to be fully elucidated (Barmada et al., 2010; Mutihac et al., 2015). Thus, factors influencing nucleocytoplasmic transport, such as the role of nuclear importins, transport-partners and effects of mutations on the TDP-43 conformation during the transit, need further investigation (Archbold et al., 2018).

### C-Terminal Fragmentation of TDP-43

The generation of C-terminal fragments of TDP-43 *via* proteolytic cleavages by the caspase and calpain proteases seems to be one of the prominent toxicity generating mechanisms, as we have discussed, previously in the “C-terminal domain” section (Zhang et al., 2007, 2009; Dormann et al., 2009; Igaz et al., 2009; Johnson et al., 2009; Yang et al., 2011; Xu, 2012; Buratti, 2015).

### Post-translational Modifications

**Figure 4:**
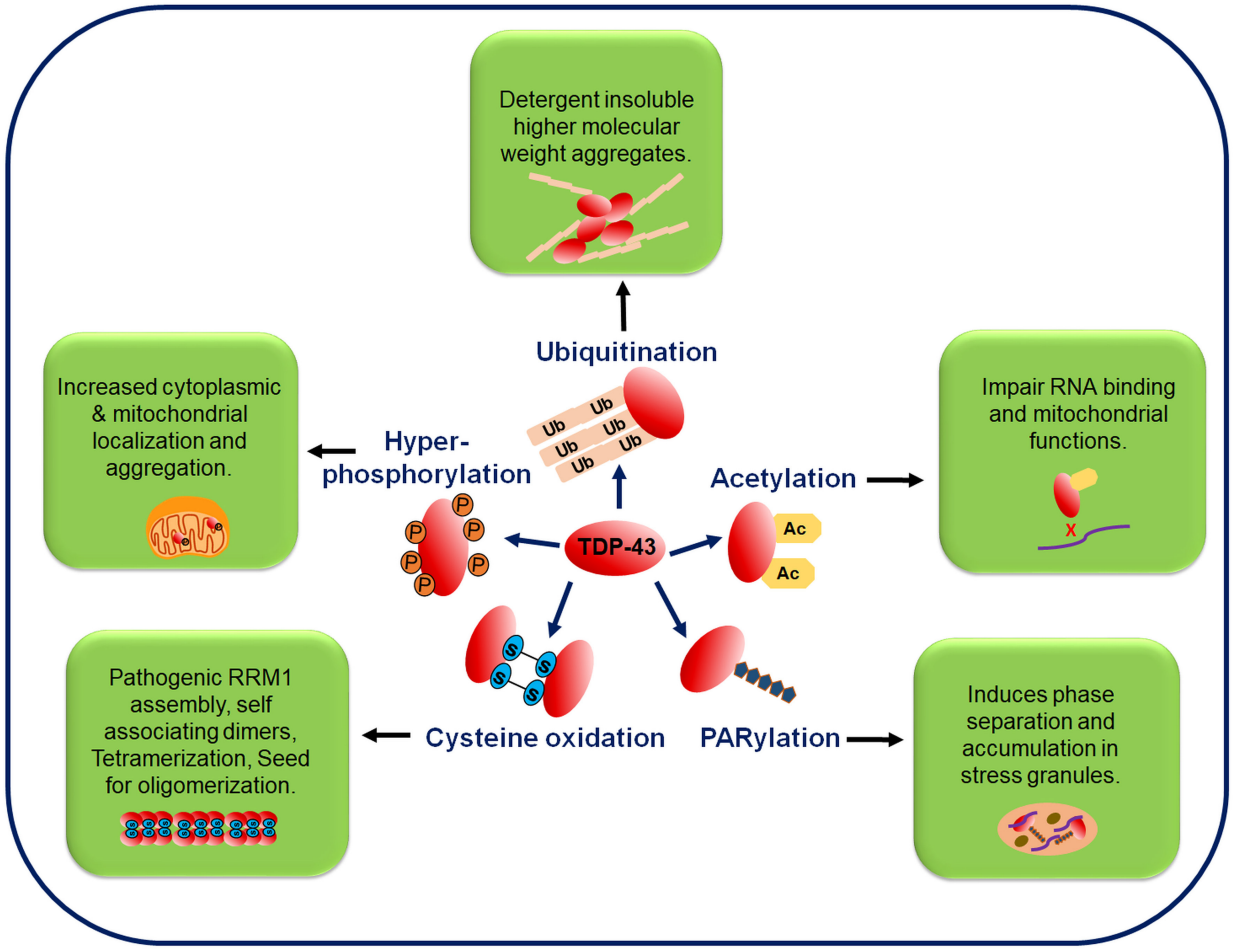
Post-translational modifications in the TDP-43 protein. TDP-43 undergoes several post-translational modifications, such as phosphorylation, ubiquitination, acetylation, PARylation, and cysteine oxidation. Phosphorylation of the full-length and C-terminal fragments of TDP-43 is a pathological hallmark of ALS and is associated with its increased cytoplasmic mislocalization. In FTLD and ALS brain inclusions, pathological TDP-43 is found in the ubiquitinated state and mutations at the ubiquitination sites decrease the TDP-43 aggregation. Acetylation promotes accumulation of the insoluble and hyper-phosphorylated TDP-43 aggregates. PARylation promotes the phase separation of TDP-43 into stress granules. Oxidative stress mediated cysteine oxidation promotes the oligomerization and aggregation. Ac, acetylation; P, phosphorylation; PARylation, poly ADP ribosylation; Ub, ubiquitination.

The two most pathologically significant common post-translational modifications (PTMs) in TDP-43 are phosphorylation and ubiquitination (Arai et al., 2006; Neumann et al., 2006, 2009; Hasegawa et al., 2008; Inukai et al., 2008). While the TDP-43's phosphorylation from TDP-43-positive inclusions in brain samples has been well-characterized owing to the availability of highly specific antibodies to detect the TDP-43's phosphorylation at different sites, the TDP-43's ubiquitination is now being investigated profusely. Recently, other PTMs like acetylation, poly ADP-ribosylation and cysteine oxidation, have also been identified from the ALS patients. Detailed characterization of the PTMs, has the potential of unearthing novel TDP-43 toxicity mechanisms in ALS (Kametani et al., 2016) (Figure 4).

#### Phosphorylation

TDP-43 has 41 serine, 15 threonine and 8 tyrosine residues, which may act as potential phosphorylation sites. The casein kinases, CK1 and CK2, have been shown to mediate phosphorylations at Ser-379, Ser-403, Ser-404, and especially Ser-409/Ser-410, which are now considered a signature of ALS pathology (Neumann et al., 2006, 2009). Another kinase, glycogen synthase kinase (GSK3) is also found to be involved in the TDP-43's phosphorylation (Sreedharan et al., 2015). The TDP-43's phosphorylation is associated with its increased cytoplasmic mislocalization and aggregation in the neuronal cells (Nonaka et al., 2009a, 2016; Barmada et al., 2010; Liachko et al., 2010; Choksi et al., 2014). Notably, distinctly phosphorylated TDP-43 inclusions have been reported in the brain cortex vs. the spinal cord cells of the ALS and FTLD patients. While affected brain cortex shows accumulation of phosphorylated C-terminal fragments, the spinal-cord cells show a predominant deposition of the phosphorylated full-length TDP-43 (Neumann et al., 2009). Antibodies developed against the phosphorylated TDP-43 have shown potential as tools for rapid detection of the TDP-43 inclusions.

#### Ubiquitination

TDP-43 has also been found in ubiquitinated state in the ALS and FTLD brain inclusions (Neumann et al., 2006, 2007b). The E3 ubiquitin ligase (Parkin) is shown to ubiquitinate TDP-43 *via* the ubiquitin lysines, K-48, and K-63. This facilitates the TDP-43's cytoplasmic accumulation into inclusions without any detectable evidence of its protein degradation (Seyfried et al., 2010; Hebron et al., 2013). The ubiquitin-conjugating enzyme UBE2E3 and ubiquitin-isopeptidase Y (UBPY) were identified, in a yeast two-hybrid screen, to interact with TDP-43 and this interaction is proposed to enhance the ubiquitination and accumulation of its insoluble high molecular weight aggregates (Hans et al., 2014). Notably, an FTLD-associated TDP-43 with K263E mutation was observed to be excessively ubiquitinated, possibly as a consequence of its misfolding due to the substitution of the positively charged lysine residue with a negatively charged aspartate residue in the RRM2 domain (Hans et al., 2014). Strikingly, Scotter et al. have demonstrated that the full-length TDP-43 aggregates are labeled by both K-48- and K-63-linked polyubiquitin chains and subsequently directed toward different fates: ubiquitin proteasomal-mediated degradation of TDP-43 for the K-48-linked polyubiquitin chains, and autophagic removal of the TDP-43 with K-63-linked polyubiquitin chains (Scotter et al., 2014). Additionally, using proteomics, several ubiquitination sites have also been identified near the TDP-43's RRM1 domain and about 35 proteins, including the RNA binding proteins rasGAP SH3 domain binding protein 1 (G3BP), poly(A)-binding protein cytoplasmic 1(PABPC1), and eukaryotic initiation factor 4A1 (eIF4A1), were found in the detergent-insoluble fractions containing the ubiquitinated TDP-43 (Dammer et al., 2012). Moreover, mutations at these ubiquitination sites were also found to decrease the TDP-43's accumulation thereby implicating the ubiquitination in modulating the TDP-43 aggregation (Dammer et al., 2012).

#### Acetylation

There are 20 lysine residues in TDP-43, some of which are prone to acetylation, such as the K-145 and K-192 (Cohen et al., 2015; Wang P. et al., 2017). Using an acetylation mimic, where lysine was mutated to glutamine residue, the TDP-43 acetylation was shown to impair RNA binding, disturb mitochondrial functions, and promote accumulation of the insoluble and hyper-phosphorylated TDP-43 aggregates in the neuronal cell cultures (Cohen et al., 2015). In another study, arsenite-induced oxidative stress could trigger the TDP-43's acetylation and formation of aggregates of ~75–250 kDa (Cohen et al., 2015; Wang P. et al., 2017). Additionally, an antibody Ac-K145 raised against the acetylation at the lysine 145 could, in fact, identify the lesions positive for acetylated TDP-43 in the ALS patient's spinal cord (Cohen et al., 2015; Wang P. et al., 2017). It remains to be examined whether any other lysines are prone to acetylation *in vivo* and if so, what are their effects on the TDP-43's aggregation. Understandably, even non-specific multi-site *in vivo*, or *in vitro* acetylation mediated through acetylating agents like aspirin, would dramatically alter the TDP-43's net charge, which can affect its aggregation propensity through electrostatic repulsions (Abdolvahabi et al., 2015; Ayyadevara et al., 2017; Prasad et al., 2018).

#### Poly ADP-Ribosylation

Poly ADP-ribosylation (or PARylation) is a post-translational modification that appears rapidly at the DNA damage sites, and has implications in cancer, cell cycle regulation, DNA repair pathways, and chromatin reorganization, etc. (Bai, 2015). Poly (ADP-ribose) polymerase (PARP) enzymes attach the ADP-ribose unit *via* an ester bond to the carboxyl group of the acidic residues, such as glutamate and aspartate on the target proteins. Polymeric PAR chains are formed when subunits are linked to one another *via* ribose-ribose bonds (Leung, 2014). The negative charge on PAR can alter the structure of the target proteins and modify the protein-DNA/RNA and the protein-protein interactions. In fact, PARylation has been found to induce phase separation of the intrinsically disordered proteins involved in ALS (Altmeyer et al., 2015). Preliminary data (Duan et al., 2018) suggests that hnRNPA1 and TDP-43 can both be PARylated and bind to PARylated proteins. The PARP enzyme, tankyrase, was shown to reduce the TDP-43's aggregation by non-covalently attaching PAR *via* PAR-binding motif present in the TDP-43 nuclear localization signal (NLS) sequence. PAR binding was found to promote the TDP-43's phase separation *in vitro* and was also shown to be essential for the TDP-43's accumulation in the stress granules in the mammalian cells and neurons (Mcgurk et al., 2018).

#### Cysteine Oxidation

In addition to the disulfide bridging for proper folding of proteins, cysteine residues also play an essential role in the maintenance of the cellular redox state. Altered cellular redox balance and oxidative stress have been proposed as contributory factors to the ALS pathology. Thus, cysteine oxidation may represent a crucial pathological pathway in ALS (Valle and Carri, 2017; Buratti, 2018). Using the *in vitro* and cell-based studies, Cohen et al. have reported that oxidative stress promotes the TDP-43's cross-linking *via* cysteine oxidation into disulfide bond formation. Among the six cysteine residues (C39, C50, C173, C175, C198, and C244) present in the TDP-43 protein, four cysteine residues at the positions 173, 175, 198, and 244, are highly conserved (in human, mouse, *Drosophila* and zebrafish) and can undergo oxidation and disulfide bond formation (Cohen et al., 2012). Importantly, the cysteine-generating ALS-linked missense mutations (G358C, S379C, and G295C) introduce additional cysteines which can potentially enhance the abnormal TDP-43 disulfide cross-linking. Notably, the inter- and intra-molecular cross-links can also result in alterations in the TDP-43's subcellular localization and solubility (Cohen et al., 2012). Structure-function analysis of the RRM1 domain has suggested that the cysteines (C-173 the C-175) in this domain, are crucial for the TDP-43's conformation and these are also involved in the pathogenic RRM1 self-assembly (Shodai et al., 2013). In another study, cysteines in the RRM2 domain (C-198 and C-244) could form self-aggregating disulfide-linked dimers upon oxidation and assembled into aggregated species (Rabdano et al., 2017). Notably, oxidation of the two N-terminal cysteines (C39 and C50) can contribute to oligomerization possibly by priming the process (seeding). Significant reduction in the oligomer formation, was observed when mutations were introduced at these positions (Bozzo et al., 2017). Another study has found that the intermolecular N-terminal cysteine disulfides result in the tetramerization of TDP-43 by formation of NTD homodimers first, and both the dimers and the tetramers inhibit the TDP-43 aggregation (Jiang et al., 2017). Cysteine residues are present in the NTD, RRM1, and RRM2 domains, can all be oxidized and result in the loss-of-function and aggregation of TDP-43 under both the *in vitro* and *in vivo* conditions. Proposedly, the oxidation-induced conformational change of RRM1 seems more crucial for the TDP-43's aggregation and the ALS pathology than the cysteine oxidation of the NTD and RRM2 domains (Chang et al., 2013). Recently, we have shown that a recombinantly purified TDP-43 C-terminal fragment, which encompasses the RRM2 domain, can spontaneously form cysteine-linked homodimers and can convert into amyloid-like aggregated species (Prasad et al., 2018).

## Aggregation of TDP-43

### Amyloid-Like Aggregation of TDP-43

Whether TDP-43 deposited in the neuronal cells has amyloid-like aggregate features, is still debated. Early reports had suggested that the filament-like structure of TDP-43 found in the ALS-affected brains do not stain with the amyloid-specific dyes, thioflavin-T (ThT) and Congo red (Neumann et al., 2006; Johnson et al., 2009). From some ALS cases, thioflavin-S (ThS)/ThT-staining amyloid aggregates have now been reported (Bigio et al., 2013; Robinson et al., 2013). Considerable interest, therefore, exists in deciphering any potentially amyloidogenic behavior of TDP-43 both *in vivo* and *in vitro*.

Recombinantly expressed full-length TDP-43 has been shown to form smooth granulo-filamentous, ThT-negative aggregates *in vitro*, similar to those found in the degenerating neurons of the ALS and FTLD patients (Johnson et al., 2009; Furukawa et al., 2011). TEM has revealed a stacking of thin fibers into thicker bundles, which also exhibit sarkosyl insolubility (Furukawa et al., 2011). Protease treatment of these full-length TDP-43 fibrillar aggregates, followed by mass spectrometry showed that the fibril core structure comprises of different C-terminal fragments spanning from the RRM1 to the C-terminal end (Furukawa et al., 2011). In yet another study, following the overexpression of TDP-43 in the bacterial cells, the TDP-43 inclusion bodies formed, were found also to be ThT-negative (Capitini et al., 2014).

However, in certain other studies, both wild-type and ALS-associated mutant TDP-43's peptides have been shown to efficiently form β-sheet-rich, ThT-positive fibrillar aggregates suggestive of their amyloid-like nature (Chen et al., 2010; Guo et al., 2011; Sun et al., 2011; Zhu et al., 2014) (Table 2). Different amyloidogenic cores for the TDP-43's aggregation have been defined from its C-terminal region, including the sequences: 286–331, 311–360, and 342–366 (Chen et al., 2010; Guo et al., 2011; Saini and Chauhan, 2011; Mompean et al., 2015; Jiang et al., 2016). The shortest peptides from TDP-43 that are shown to form amyloid-like aggregates are DLII (247–250) and NFGAF (312–316), which bear resemblance to the amyloidogenic core sequence of the human islet amyloid polypeptide (IAPP) (Furukawa et al., 2011; Saini and Chauhan, 2011, 2014; Prasad et al., 2016). Notably, TDP-43 peptides containing the ALS-linked mutations like A315T and G335D have been found to enhance amyloid-like aggregation with self-seeding and cross-seeding abilities (Guo et al., 2011; Jiang et al., 2016). It has been argued that the familial mutations in the C-terminal region increase the propensity of the short α-helices toward β-sheet structural transition (Sun and Chakrabartty, 2017).

High resolution structures have been obtained of the amyloidogenic peptides from the RRM2 domain and the low complexity domain (LCD) of TDP-43, which could adopt the characteristic amyloid steric zipper structures (Guenther et al., 2018a,b). An RRM2 peptide, aa 247–257, was shown to form distinct types of amyloid aggregates that fit into different classes of steric zipper structures. This polymorphic ability was attributed to its ability to adopt different backbone conformations (Guenther et al., 2018b). Furthermore, a peptide from the LCD region, aa 312–317, and its ALS-linked mutant variants, A315E and A315T, were also shown to form kinked β-sheet structures which promote the formation of phase separated droplets and hydrogels, unlike several other peptides of this LCD region (Guenther et al., 2018a).

Alike to as previously reported for the Amyloid β (Aβ)-42 peptide's amyloid aggregation, a low net charge on the TDP-43 protein decreases its solubility and improves its aggregation, whereas, with high net charge the electrostatic repulsions dominate, which can impede the aggregation of TDP-43 (Mompeán et al., 2016a). We have, in fact, recently explored the *in vitro* amyloidogenic aggregation of a C-terminal fragment (aa 193–414) of TDP-43 in the presence of different Hofmeister series anions. We found that kosmotropic anions greatly accelerate whereas the chaotropic anions impede its amyloid-like aggregation rates (Prasad et al., 2018). Amyloid fibril morphological features also varied in the presence of the kosmotropic vs. the chaotropic anions. Furthermore, *in vitro* aspirin-mediated non-specific lysine acetylations, which would mask the lysine's charges, significantly reduced the TDP-43's C-terminal fragment's amyloid-like aggregation (Prasad et al., 2018).

### Physiological vs. Pathological Oligomerization of TDP-43

For several neurodegenerative diseases like the Alzheimer's, Parkinson's and prion diseases, the neuronal cytotoxicity is proposedly exerted through oligomeric forms of the aggregating proteins/peptides (Kayed et al., 2003; Haass and Selkoe, 2007). Recently, several studies have also examined TDP-43's oligomerization and its potential neurotoxic properties (Table 2). Evidence suggests that in the normal brain, TDP-43 exists in dimeric form predominantly in the neuronal cell nucleus (Kuo et al., 2009; Shiina et al., 2010; Afroz et al., 2017). The NTD region, especially its first 10 amino acids, appear to be indispensable for the dimerization (Chang et al., 2012; Zhang Y. J. et al., 2013; Mompean et al., 2017). Recently, cross-linking experiments have revealed that in the normal human brain, TDP-43 can exist not only as dimers, but rather in a spectrum of oligomeric species viz. dimers, trimers, tetramers and multimers (Afroz et al., 2017). This oligomerization is proposed to be important for the TDP-43's functional roles in the RNA binding, probably by its increased affinity and specificity for its RNA targets, and/or *via* optimal recruitment of the other RNA splicing factors.

In contrast, pathological forms of TDP-43 oligomers have also been reported (Table 2), which may be structurally distinct from the nuclear TDP-43 oligomers. Shiina et al. have reported that the N-terminal region (aa 3–183) acts as an intermolecular interacting domain in an 86 kDa dimeric form of TDP-43 overexpressed in the cells. Thus, they have proposed that the dimeric TDP-43 may seed the formation of the pathological higher molecular weight TDP-43 aggregates (Shiina et al., 2010). Indeed, expression of a tandem TDP-43 construct expressing TDP-43 repeat as an 86 kDa protein in the HEK293 cells, induced the accumulation of TDP-43 aggregates. Furthermore, an 86 kDa species was also observed in an immunoblot of extracts from the deceased ALS brains (Shiina et al., 2010).

Fang et al. have reported that the full-length TDP-43 forms spheroidal and ring-like oligomeric structures with cytotoxicity to the neuronal cells (Fang et al., 2014). Following purification of recombinantly expressed full-length TDP-43 by size exclusion chromatography, DLS and TEM analyses have shown that the fractions containing oligomeric TDP-43 have a size distribution of 40–400 nm. The TDP-43 oligomers also manifest a propensity to cross-seed Aβ-42 peptide thereby demonstrating a structural inter-convertibility among the common amyloid oligomeric structures (Kayed et al., 2003; Fang et al., 2014). TEM analysis of gold immunolabelled FTLD-TDP brain fractions has revealed TDP-43 oligomers with a diameter of ~50 nm (Fang et al., 2014; Kao et al., 2015). Furthermore, polyclonal antibodies raised against the TDP-43 oligomers (TDP-O) could not only detect the oligomeric aggregates obtained *in vitro*, but more importantly also the oligomers from the brain sections of the TDP-43 mice model and also those from the FTLD-TDP affected patients. This is a step forward toward the development of TDP-43 oligomer detection as a biomarker for ALS.

In a recent study, beneficial forms of TDP-43 oligomers have been identified in the skeletal muscles (Vogler et al., 2018). These SDS-resistant oligomers were found to be distinct from those observed in stress granules, and were termed as myo-granules. Furthermore, the myo-granules exhibited amyloid-like characteristics. X-ray diffraction of the lyophilized myo-granules showed a diffraction pattern with a 4.8 Å reflection indicating a β-sheet-rich structure, however they lacked a 10 Å reflection which suggests that these myo-granules lack the typical cross β-sheet arrangement. The TDP-43 myo-granules seem functionally significant as they contain the mRNAs that encode for proteins involved in the formation of sarcomeres (Becker and Gitler, 2018; Vogler et al., 2018).

### Prion-Like Behavior of TDP-43 Aggregates

The fatal human neurodegenerative diseases Creutzfeldt-Jakob Disease (CJD) and Kuru involve deposition of the infectious prion protein PrP in aggregated amyloid-like conformation in the affected brains (Aguzzi et al., 2008; Aguzzi and Calella, 2009). Prions were first proposed by Stanley Prusiner to be novel “protein-only” infectious agents (Prusiner, 1982). Fungi, such as yeast and *Podospora* have also been found to harbor prion-like elements (Wickner, 1994; Derkatch et al., 2001; Maddelein et al., 2002; Patel et al., 2009; Liebman and Chernoff, 2012). Several of the fungal prions have been vividly shown to infect in a “protein only” fashion (King and Diaz-Avalos, 2004; Tanaka et al., 2004; Patel and Liebman, 2007). The transmissibility of the infectious prion aggregates is attributed to their exceptional protease and detergent resistance and to their ability to propagate from cell-to-cell and organism-to-organism by “seeding” to induce more pathological aggregates (Caughey et al., 2009; Cobb and Surewicz, 2009). In fact, several yeast prions can also influence the aggregation and/or toxicity of certain human amyloidogenic proteins, such as poly-glutamine, transthyretin and TDP-43 etc., proposedly *via* heterologous cross-seeding or by influencing the chaperone availability (Derkatch et al., 2001; Meriin et al., 2002; Park et al., 2017; Verma et al., 2018).

Accumulating evidence suggests that several other proteins previously considered as non-prion proteins, including Aβ-42, α-synuclein, and TDP-43 etc., can exhibit prion-like behavior both *in vitro* and in the disease models (Brundin et al., 2010; Hock and Polymenidou, 2016). In the case of TDP-43, Furukawa et al. have reported that the transduction of pre-formed, sarkosyl-insoluble, fibrillar aggregates of the recombinantly expressed full-length TDP-43 into the HEK293T cells expressing TDP-43, induces the aggregation of the endogenous TDP-43 into detergent-insoluble and ubiquitinated inclusions, similar to those observed in the ALS patients (Furukawa et al., 2011). In another pivotal study, Nonaka et al. have identified different strains of the TDP-43 aggregates from the ALS/FTLD diseased brains (Nonaka et al., 2013). When these TDP-43 aggregates were introduced into SH-SY5Y human neuroblastoma cells expressing TDP-43, seed-dependent formation of the insoluble TDP-43 inclusions was observed, resembling the pathological profiles of the parent TDP-43 seeds used. Also, the TDP-43 aggregates could be propagated between the cells over serial passages thereby further supporting their prion-like behavior. Additionally, the seeding ability of the insoluble TDP-43 was unaffected by heat or proteinase treatment, but was abrogated by formic acid, indicating that the β-sheet structure of these aggregates is important for the seeding capability (Nonaka et al., 2013).

In another study, oligomeric TDP-43 obtained from sorbitol-induced osmotic stress in the HEK293 cells, as well as oligomers derived from the ALS brain lysates, were found to show seeding ability and intercellular transmission *via* microvesicles/exosomes (Feiler et al., 2015). Additionally, Smethurst et al. have identified a diverse range of TDP-43 inclusion bodies, such as skeins, dot-like, and granular etc., upon seeding with the pathological TDP-43 aggregates from different ALS patient's brains into the cells expressing full-length TDP-43, possibly indicating strain-like propagation characteristic of prions. In fact, repeated inoculation with the TDP-43-containing insoluble fractions showed a consistent increase in the accumulated TDP-43 thereby supporting prion-like seeded aggregation and a capability of intercellular transmission (Smethurst et al., 2016).

In a recent study, Ishii et al. have demonstrated a time-course microscopy of formation of phosphorylated and ubiquitinated TDP-43 aggregates in electron-dense granules and intercellular spreading of these TDP-43-positive granules into the cells subjected to MG-132-induced stress and expressing wild-type and C-terminal region of TDP-43 (Ishii et al., 2017). In a separate study, 40 amino acid long peptides (spanning aa 274–313 and 314–353) could form amyloid-like fibrils *in vitro* and transduction of these amyloid fibrils induced inclusion body formations that contained phosphorylated C-terminal TDP-43 fragments in the SH-SY5Y cells expressing the wild-type TDP-43 (Shimonaka et al., 2016). Furthermore, prion strain-like behavior was also observed when trypsin digestion of the TDP-43-positive, sarkosyl-insoluble fractions, was found to show different TDP-43 band patterns from the 274–313 and 314–353 fibril-treated cells, indicating template-dependent aggregation (Shimonaka et al., 2016). The TDP-43 oligomers involved in the seeded aggregation and prion-like transmission can be therapeutic targets hence how they propagate need detailed elucidation.

### Phase Separation of TDP-43

**Figure 5:**
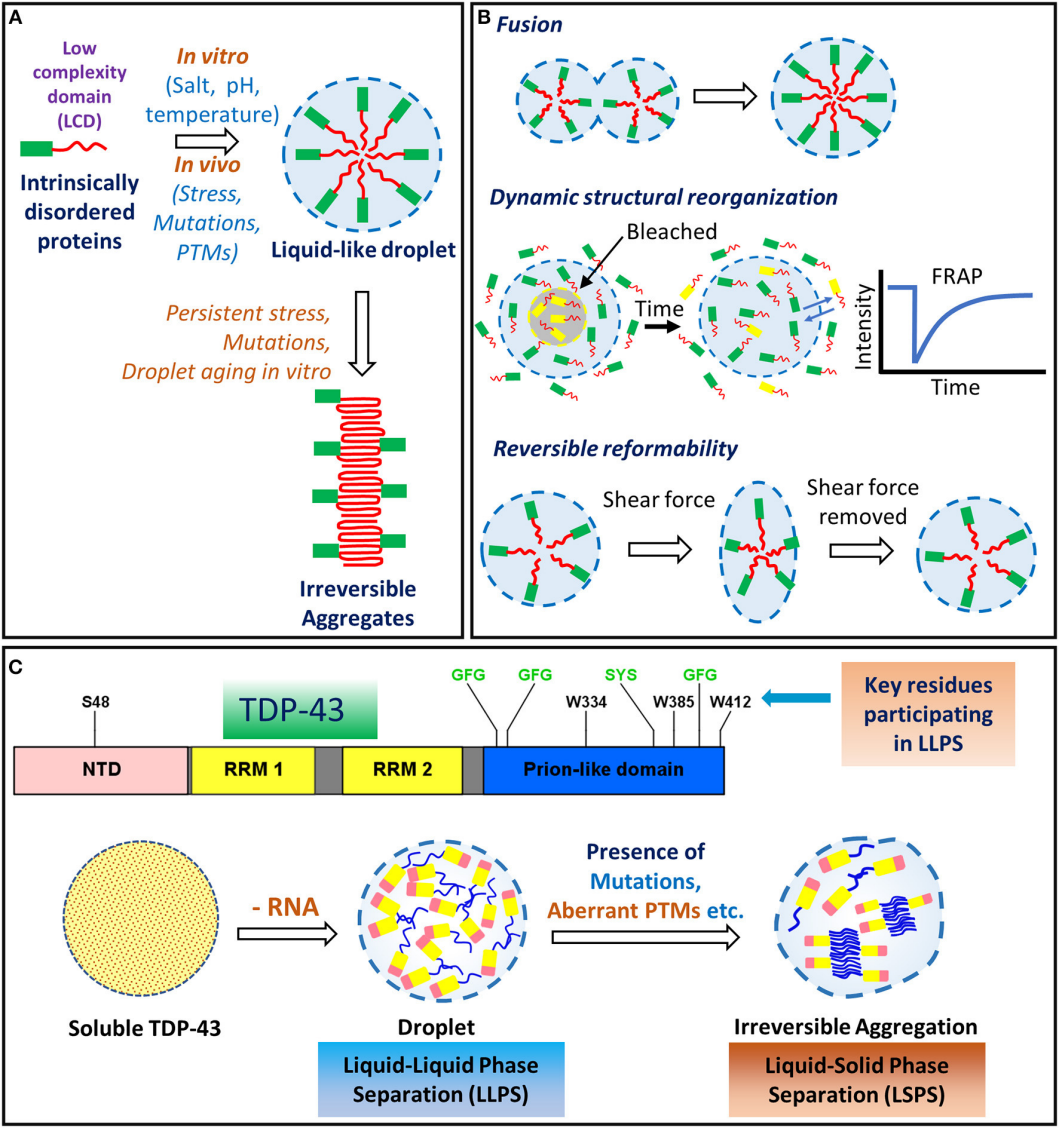
Liquid-liquid phase separation (LLPS) and liquid-solid phase separation (LSPS) of TDP-43. **(A)** Proteins containing low complexity/prion-like domains undergo phase-separation into membrane-less, spherical compartments, often aided by the presence of salt, pH changes or temperature changes. Persistent stress, mutations and droplet-aging, might induce irreversible aggregation into pathological structures, such as the amyloid-like aggregates. **(B)** Liquid droplet-like properties are manifested by the intrinsically disordered proteins, such as: the ability of the smaller droplets to freely fuse into a larger droplet; transient intermolecular interactions allowing the dynamic rearrangement of the internal structural components; and reversible reformability upon removal of the external shear forces. **(C)** Liquid-liquid phase separation (LLPS) of TDP-43 is influenced by both hydrophilic and hydrophobic residues. The (G/S)-(F/Y)-(G/S) motifs (highlighted in green) promote the phase separation through transient interactions in several intrinsically disordered proteins (Li et al., 2018). The tryptophan residues promote LLPS by hydrophobic interactions (Li et al., 2018). Depletion of the TDP-43's interactions with RNA molecules, upon high protein: RNA ratio, can lead to irreversible aggregation *via* Liquid-solid phase separation (LSPS) (Maharana et al., 2018). ALS-linked mutations are also proposed to lead to the formation of the irreversible aggregates. FRAP, fluorescence recovery after photobleaching; LCD, Low complexity domain; LLPS, liquid-liquid phase separation; LSPS, liquid-solid phase separation; NTD, N-terminal domain; PTM, post-translational modification; RRM, RNA recognition motif.

An increasingly recognized process being implicated in several neurodegenerative diseases is the formation of membraneless liquid droplet-like organelles by the proteins containing prion-like domains through a process called liquid-liquid phase separation (LLPS) (Figure 5) (Shin and Brangwynne, 2017). Several RNA binding proteins like TDP-43, FUS, hnRNPA1 and hnRNPA2/B1 etc., contain intrinsically disordered regions and can undergo phase separation through transient intermolecular interactions (Burke et al., 2015; Lin et al., 2015; Molliex et al., 2015; Patel et al., 2015; Conicella et al., 2016; Batlle et al., 2017; Gopal et al., 2017; Li et al., 2017; Sun and Chakrabartty, 2017; Uversky, 2017). Proteins with a prion-like low complexity domain (LCD), exhibit in this region, an over-representation of polar and charged amino acids including arginine, lysine, glutamine, serine, glutamic acid and occasionally glycine, alanine and proline with interspersed aromatic residues, particularly tyrosine and phenylalanine (Shin and Brangwynne, 2017). LLPS behavior appears to be driven by transient intermolecular interactions, such as the hydrophobic, cation-pi and pi-pi interactions, as well as the charge patterning of the polar and charged amino acids in the prion-like LCD domains (Shin and Brangwynne, 2017; Simon et al., 2017).

Phase-separated droplets of the ALS-linked FUS mutants were found to display a propensity to mature into amyloid-like fibrillar aggregates (Patel et al., 2015). Hence, LLPS appears to be an immense risk factor as the transient localization of the intrinsically disordered proteins into the droplets under stress conditions, possess the peril of their conformational transitions within the liquid compartments into pathological irreversible aggregates. The phase separation behavior of the RNA binding proteins, seems closely associated with their propensity to form stress granules (Molliex et al., 2015; Protter and Parker, 2016; Riback et al., 2017).

In one study, although mutant TDP-43 droplets did show irregular morphologies, the ThT staining was not indicative of amyloid-like features (Conicella et al., 2016). Conicella et al. have reported that prion-like TDP-43's C-terminal region (aa 276–414) undergoes phase separation *in vitro* in the presence of salt and RNA. Interestingly, certain ALS-associated TDP-43 mutations, such as A321G, Q331K, and M337V, have been found to decrease the phase separation ability and increase the propensity to aggregate with irregular morphology (Conicella et al., 2016). Structural analysis has shown that a tryptophan residue, W334, in the α-helical segment (aa: 320–340) is crucial for the TDP-43's prion-like domain's phase separation (Li et al., 2017, 2018). Wang et al. suggest that a phosphomimetic substitution at S48 in the NTD disrupts the TDP-43's LLPS and decreases the NTD's polymerization, and thus, it is a conserved phosphorylation site found to be phosphorylated at low levels in the ALS *in vivo* models (Wang et al., 2018). Strikingly, the poly(ADP-ribose) polymerase, tankyrase, was found to modify TDP-43 by adding negatively charged poly(ADP-ribose) polymer to its nuclear localization signal sequence, which promoted LLPS and facilitated the TDP-43's accumulation into stress granules in the neuronal cells (Mcgurk et al., 2018).

Recently, Gopal et al. have shown that TDP-43 containing RNP transport granule, in the axonal cells, display droplet-like properties, such as spherical shape, fusion, deformability upon shear force, rapid internal TDP-43 redistribution and sensitivity to disruption of the weak hydrophobic interactions by 1,6-hexanediol treatment. Also, ALS-linked TDP-43 mutations like M337V and G298S were found to display increased granule viscosity and disrupted axonal transport functions (Gopal et al., 2017). Strikingly, depletion of the TDP-43's interaction with the RNA molecules in cells, upon high protein:RNA ratio, was recently found to cause TDP-43's irreversible aggregation *via* liquid-solid phase separation (LSPS) (Maharana et al., 2018). Thus, finding modulators of the phase separation may have tremendous therapeutic potential.

## Emerging Mechanisms of TDP-43-Induced Cytotoxicity

### Dysregulation of TDP-43 Protein Turnover

**Figure 6:**
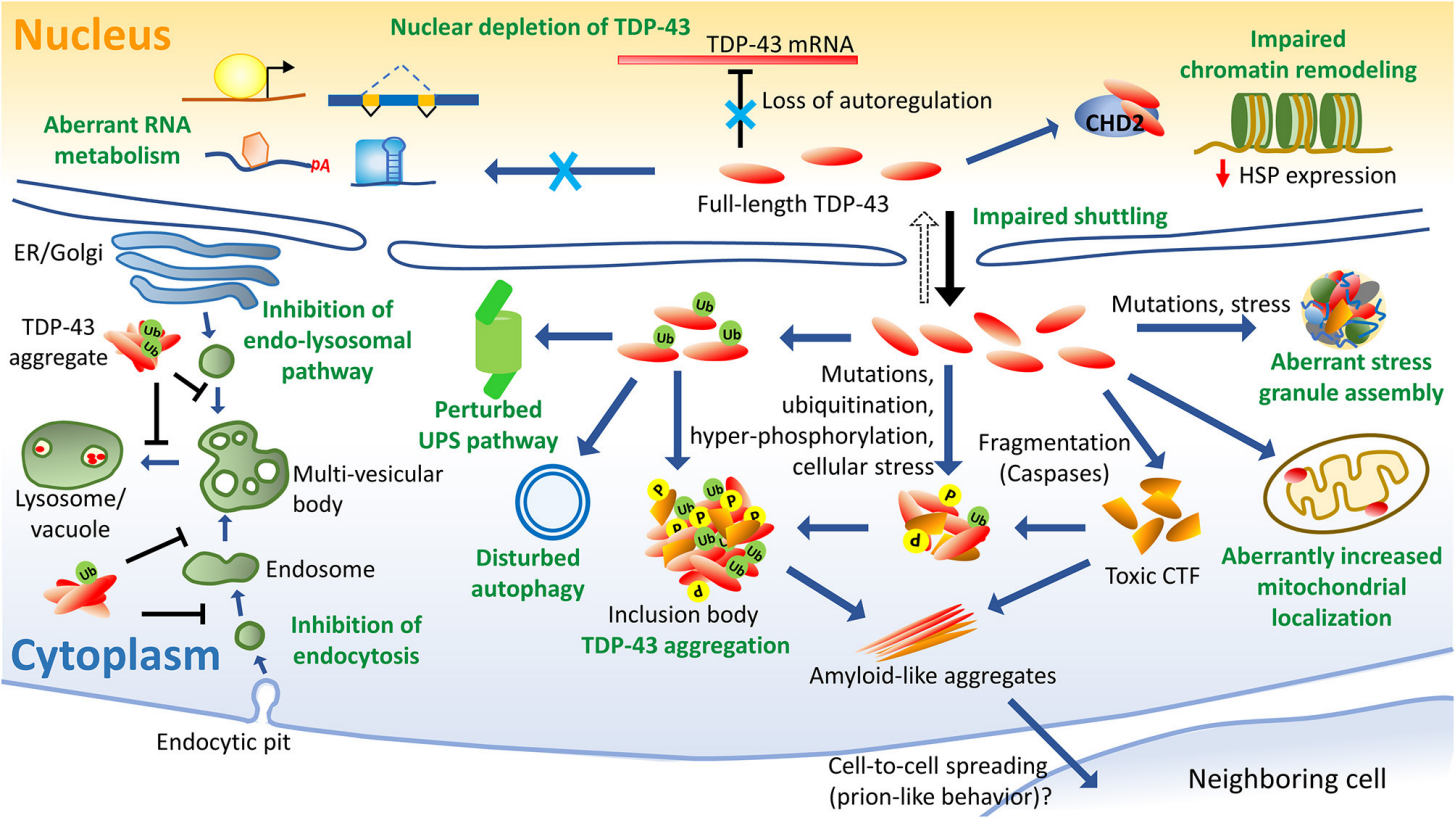
Schematics of TDP-43-induced pathology. Several aspects of TDP-43-linked cellular dysfunctions have been identified in ALS, such as nuclear depletion which leads to aberrant RNA metabolism and a loss of autoregulation of TDP-43 levels. Cytoplasmic accumulation of the hyper-phosphorylated and ubiquitinated TDP-43 are ALS disease hallmarks. Fragmentation of TDP-43 leads to the formation of toxic and aggregation-prone C-terminal fragments (CTFs). TDP-43 mutations can lead to abnormal stress granule assembly and release. Aberrantly increased mitochondrial localization of TDP-43 impairs its function. TDP-43 is also associated with the misregulated autophagy and proteosomal processes. TDP-43 expression perturbs the endocytosis process possibly by altering the expression of key endocytic components. Also, the TDP-43 aggregates have been identified as an inhibitor of the endolysosomal pathway. TDP-43 interacts with chromatin remodeling protein CHD2 and perturbs the chromatin dynamics which prevents the expressions of heat shock proteins. Prion-like inter-cellular propagation of detergent-resistant, β-sheet-rich aggregates of TDP-43, has also been demonstrated in the neuronal cell models. CHD2, chromodomain helicase DNA binding protein 2; CTF, C-terminal fragments; ER, endoplasmic reticulum; HSP, heat shock protein; P, phosphorylation; Ub, ubiquitination; UPS, ubiquitin-proteasome system.

Protein homeostasis in a cell is maintained *via* ubiquitin-proteasome system (UPS), autophagy and ER stress-activated unfolded protein response (UPR). Abnormal turnover of TDP-43 caused by mislocalization and aggregation appears as a key event for ALS and aberrations in the neuronal proteostasis have been identified in ALS (Braun, 2015; Budini et al., 2017; Ramesh and Pandey, 2017) (Figure 6).

TDP-43 has been found to be involved in the regulation of autophagy by associating with the mRNA of a key autophagy-associated protein ATG7 (autophagy related 7), however, some of the ALS-linked TDP-43 mutations can abolish its ATG7 mRNA binding ability (Bose et al., 2011). TDP-43 can also affect the localization of the transcription factor TFEB (transcription factor EB) which regulates the expression of several autophagy lysosomal pathway proteins in the neuronal cells (Xia et al., 2016). Inclusion bodies positive for autophagy markers like LC3 and p62/SQSTM1, have been identified in the ALS and FTLD patients' spinal cords suggesting the involvement of autophagy in the ALS disease progression (King et al., 2010a; Budini et al., 2017). The ALS-associated mutations in *UBQLN2* cause impaired autophagy and induce increased overall TDP-43 levels and promote the TDP-43 aggregation in the neuronal cells (Osaka et al., 2016). Araki et al. have found that the disease-associated TDP-43 mutants like G298S and A382T, are more rapidly turned over than the wild-type protein, through the ubiquitin-proteasome system, thus highlighting the pathological relevance of the TDP-43 proteolysis and clearance (Araki et al., 2014).

The role of autophagy in rescuing TDP-43-associated toxicity might be a complex process as suggested by conflicting data showing that autophagy can either accelerate or slow down disease progression (Barmada et al., 2014). In a systematic genetic screen in the yeast cells expressing TDP-43, it was found that the vacuolar fusion machinery and the endo-lysosomal pathways are critical for the TDP-43 clearance and for maintaining the cell survival. Strikingly, the autophagy pathway that contributed to the TDP-43 clearance was also found to increase cytotoxicity (Leibiger et al., 2018). Filimonenko et al. have reported that TDP-43 accumulation increases in the cells with defective autophagy processes. The endosomal sorting complexes required for transport (ESCRT) are important proteins involved in the autophagy pathway. Depletion of ESCRT subunits results in the formation of multivesicular bodies (MVBs) with abnormal morphology. In ESCRT-depleted cells, TDP-43 was found to accumulate in the ubiquitin-positive inclusions (Filimonenko et al., 2007).

The full-length TDP-43 and its fragments, are also known ubiquitin substrates that are directed for degradation either through the ubiquitin-proteasome system (UPS) or autophagy. Early studies suggested that the soluble as well as the aggregated TDP-43 are cleared by both the ubiquitin-proteasome system (UPS) and autophagy (Urushitani et al., 2009; Wang et al., 2010; Zhang et al., 2010). Recently, Scotter et al. have shown that the soluble TDP-43 is mainly degraded by the ubiquitin-proteasome system (UPS), whereas the cytotoxic aggregated forms of TDP-43, are preferentially removed through autophagy (Scotter et al., 2014). Barmada et al. have identified potent compounds from a pharmacophore library that can significantly stimulate neuronal autophagy and enhance TDP-43 turnover, thereby improving the growths of the primary neurons, human iPSC-derived neurons and astrocytes (Barmada et al., 2014).

### Impairment of Endocytosis

Defective endocytosis might be a contributory factor for the TDP-43's toxicity in ALS. Abnormal levels of TDP-43 inhibit endocytosis by co-localizing with the endocytosis-associated proteins in the yeast cells and cellular models, and such co-localization was also observed in an ALS patient's frontal cortex tissue (Liu et al., 2017). Impaired endocytosis has been linked with increased TDP-43 aggregation, while enhancing endocytosis was found to reverse the TDP-43 toxicity and the motor neuron dysfunction (Liu et al., 2017). In another study, TDP-43 knockdown was found to specifically reduce the number and motilities of recycling endosomes in the human iPSC-derived neurons, whereas TDP-43 overexpression caused the opposite effect (Schwenk et al., 2016). Furthermore, TDP-43 knockdown was also seen to affect the dendrite growth by decreasing the expression of the cell surface receptors crucial for neuronal growth and survival (Schwenk et al., 2016). Leibiger et al. have also shown that the endocytosis and the endo-lysosomal pathway are markedly disturbed by TDP-43 expression (Figure 6). The endo-lysosomal pathway could also contribute to TDP-43 clearance independent of autophagy. The importance of the endo-lysosomal pathway in TDP-43 pathology is highlighted by the observation that certain ALS-associated genes encode for components of this pathway, e.g., charged multivesicular body protein 2B (CHMP2B) (Leibiger et al., 2018).

### Aberrantly Increased Localization of TDP-43 to Mitochondria and Associated Toxicity

**Figure 7:**
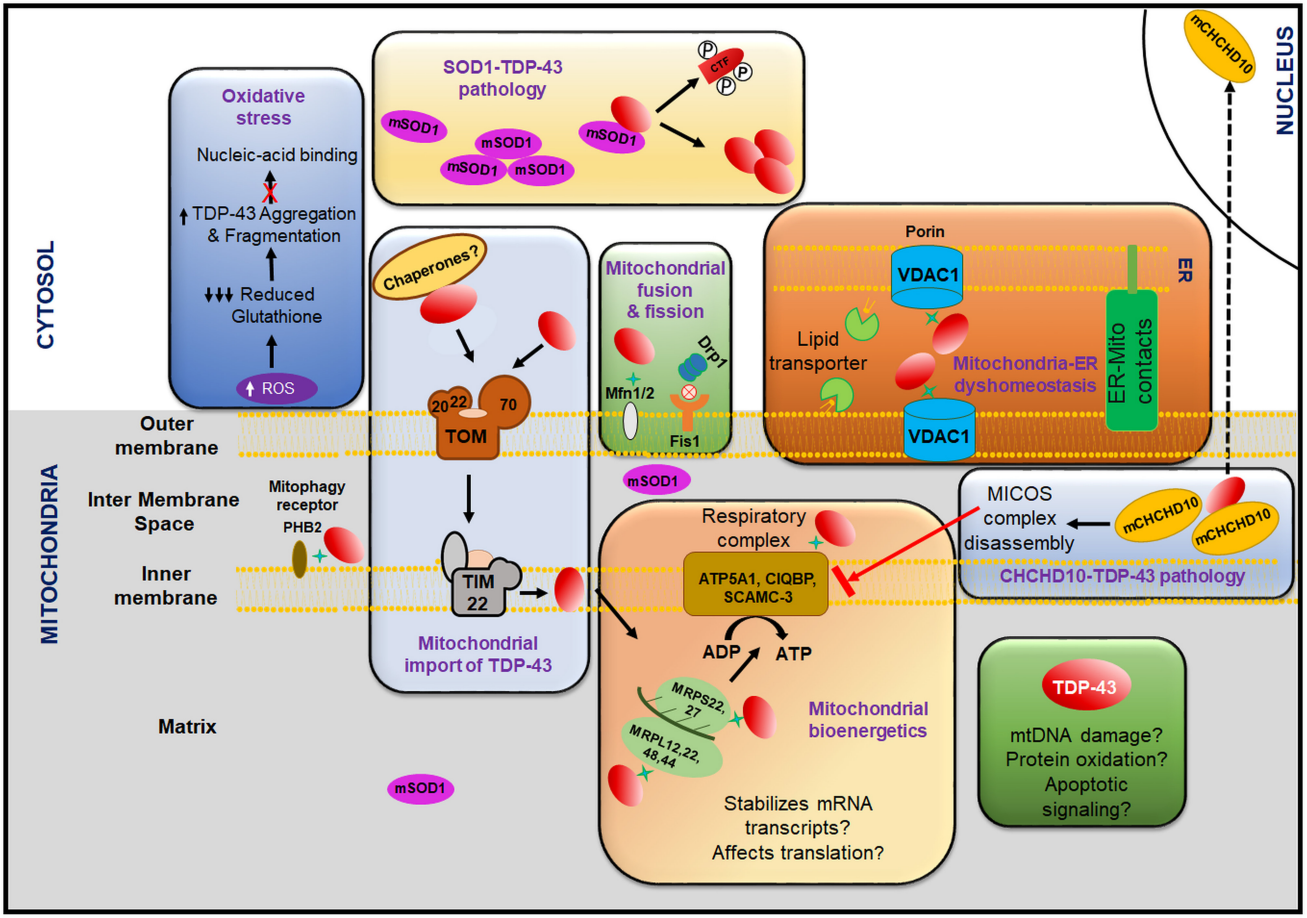
Role of mitochondria in the TDP-43 pathology. TDP-43 mediated dysfunction of the mitochondria leads to increase in the production of ROS that causes decline in the reduced glutathione levels which in turn can increase the aggregation of TDP-43 and also inhibit TDP-43 from binding to the nucleic acid. Mutant SOD1 can cause cytoplasmic mislocalization, fragmentation, phosphorylation and aggregation of TDP-43. Inhibition of the interaction of the mitochondrial fission proteins Drp1 and Fis1 greatly reduces the mitochondrial dysfunction caused by the TDP-43 overexpression/aggregation. TDP-43 also disrupts the ER-mitochondrial contacts which can have potential implications to the calcium signaling, ATP production and lipid transport. TDP-43 is imported into mitochondria *via* the outer membrane complex (TOM70) and across the inner membrane *via* TIM22. Several factors like chaperones and mitochondrial membrane potential might play a role in the TDP-43 import. After internalization, TDP-43 is found to interact with several proteins involved in the mitochondrial translation machinery (MRPS22, 27 and MRPL12, 22, 48, 44) and the mitochondrial respiratory complex (ATPA, CIQBP, SCAMC-3). TDP-43 also perturbs the translation of ND3/6 of the respiratory complex I and thus severely impairs the mitochondrial bioenergetics and reduces the ATP production. TDP-43 overexpression alters the CHCHD10 localization from the mitochondria to the nucleus and the loss-of-function mutations in *CHCHD10* are associated with MICOS complex disassembly and may negatively regulate the assembly of the respiratory complex. TDP-43 also interacts with other crucial mitochondrial proteins including the mitochondrial fusion protein Mfn2 and the mitophagy receptor PHB2. TDP-43 is depicted here by the red oval structure. TDP-43 interaction with the mitochondrial proteins are depicted *via* green star. Inhibition is denoted by red cross mark. ATPA, ATP synthase subunit A; CHCHD10, coiled-coil-helix-coiled-coil-helix domain containing 10; CIQBP, complement component 1Q binding protein; CTF, C-terminal fragments; Drp1, dynamin related protein 1; ER-Mito contacts, endoplasmic reticulum (ER)-mitochondria contacts; Fis1, fission 1 (mitochondrial); Mfn2, mitofusin-2; MICOS, mitochondrial contact site and cristae organizing system; MRPL, mitochondrial ribosomal protein (large subunit); MRPS, mitochondrial ribosomal protein (small subunit); ND, NADH dehydrogenase; P, phosphorylation; PHB2, prohibitin-2; ROS, reactive oxygen species; SCAMC-3, small calcium-binding mitochondrial carrier protein 3; SOD1, superoxide dismutase 1; TIM, translocase of inner membrane; TOM, translocase of outer membrane.

Neuronal vulnerability to the mis-localized and/or aggregated proteins, has often been found to be mediated *via* dysfunctional mitochondria (Saxena and Caroni, 2011). Post-mitotic neurons have high demands for ATP for the maintenance of the ionic gradients across cell membranes and for the intracellular communication (Kann and Kovacs, 2007; Verkhratsky et al., 2014). Hence, defects in the mitochondrial transport, mitochondrial length, intracellular Ca^2+^ levels, mitochondrial respiration and ATP production, can severely impede the proper functioning of neurons and can accelerate neurodegeneration [reviewed in (Lin and Beal, 2006; Reddy, 2009; Johri and Beal, 2012; Lezi and Swerdlow, 2012; Smith et al., 2017)]. Mitochondrial dysfunction has been recorded in both *in vivo* and *in vitro* models expressing the wild-type TDP-43 or its mutants, thus implicating the mitochondria as a route for mediating the TDP-43 toxicity (Braun et al., 2011; Wang W. et al., 2013; Stribl et al., 2014) (Figure 7).

Over-expression of TDP-43, or its mutants, in the primary motor neurons has been found to result in reduction of the mitochondrial length and impaired mitochondrial movement which could be reversed upon the co-expression of the mitochondrial fusion protein, mitofusin-2 (Mfn2) (Wang W. et al., 2013). Also, in the mouse model, expression of the mutant TDP-43 resulted in abnormal mitochondrial transport and distribution (Magrane et al., 2014). Furthermore, expression of TDP-43 in the fly model was documented to enhance mitochondrial fission and fragmentation thereby suggesting an impairment of the mitochondrial dynamics (Altanbyek et al., 2016). In the yeast model, TDP-43 expression was found to cause increased oxidative stress and formation of peri-mitochondrial TDP-43 aggregates. Importantly, presence of functional mitochondria was observed to exacerbate the deleterious effects of TDP-43 hence implicating mitochondria as a target for mediating the TDP-43 toxicity (Braun et al., 2011; Braun, 2012). Recently, TDP-43 and its disease-associated mutants have been found to significantly enhance the mitochondrial abnormalities across various models thereby reflecting the mitochondrial dysfunction observed in the ALS patients (Wang W. et al., 2013; Wang et al., 2016).

Mitochondria are the known primary sites for the production of reactive oxygen species (ROS) and also as the major target of the ROS-induced damage. Protein oxidation/carbonylation, lipid peroxidation, depletion of anti-oxidants like glutathione, increase in the intracellular free iron content and damage to DNA, are widely used markers of oxidative stress (Farrugia and Balzan, 2012) (Figure 7). Strikingly, mutant TDP-43 was found to induce oxidative damage and cause increased accumulation of the anti-oxidant response modulator, Nrf2, in the nucleus (Duan et al., 2010). Subsequently, it was also found that, although TDP-43 increased the localization of Nrf2 to the nucleus, the total expression of Nrf2 was, in fact, markedly decreased and the Nrf2/ARE pathway was impaired in the NSC-34 cell lines resulting in reduced neurites and increased lipid peroxidation products (Tian et al., 2017). Also, expression of TDP-43 in *Drosophila*, was recorded to increase the levels of protein carbonylation and glutathione S-transferase D1 (Carri et al., 2015; Zhan et al., 2015). Recently, by developing an easy red/white color assay, we have confirmed that the TDP-43 aggregation also induces oxidative stress in the yeast TDP-43 aggregation model (Bharathi et al., 2016, 2017).

TDP-43 aggregation and oxidative stress seem to mutually abet each other. Depletion of glutathione using ethacrynic acid increases the insolubility of TDP-43 and also promotes the fragmentation of TDP-43 in the primary cortical neurons (Iguchi et al., 2012) (Figure 7). Consistent with this, modification of TDP-43 with the product of lipid peroxidation, 4-hydroxynonenal, has been observed to result in a considerable increase in the insolubility and cytosolic localization of TDP-43 in COS-7 cells (Kabuta et al., 2015). Recently, increasing intracellular reduced glutathione (GSH) by treating the TDP-43 mutant expressing cells with GSH monoethyl ether, has been shown to reduce the aggregate formation, ROS generation and cell death (Chen et al., 2018). Furthermore, on subjecting the TDP-43 expressing cells to various oxidants, it was found that the cysteine oxidation and disulfide bond formation promoted the aggregation of TDP-43 (Cohen et al., 2012). In agreement, oxidation of cysteine residues in the RRM1 domain enhanced protein aggregation and inhibited the nucleic-acid binding ability of TDP-43 (Chang et al., 2013). In summary, the interplay of TDP-43 aggregation and oxidative stress instigate the toxicity of TDP-43 as well as its deleterious effects on the mitochondria.

Interestingly, superoxide dismutase 1 (SOD1), which is also implicated in ALS pathology, is transported to the mitochondria *via* translocase of the outer membrane (TOM) complex, even though SOD1 lacks a mitochondrial localization signal. Mutant SOD1 accumulates in the intermembrane space (IMS) and matrix of mitochondria and elicits toxicity (Zeineddine et al., 2017). Misfolded SOD1 also aggregates on the outer mitochondrial membrane (OMM) and is involved in mitochondria dependent apoptosis. Of note, the addition of exogenous mutant SOD1 aggregates has been reported to cause cytoplasmic mislocalization of TDP-43 and enhance its aggregation (Zeineddine et al., 2017) (Figure 7). Also mutant SOD1 expression has been found to increase the C-terminal fragmentation and phosphorylation of TDP-43 and the interaction of the mutant SOD1 with TDP-43 fragments has been speculated to mediate toxicity *via* apoptosis (Jeon et al., 2018).

The mechanistic details of how TDP-43 damages the function of mitochondria are now being uncovered. Expression of mutant TDP-43 disrupts the ER-mitochondrial connection by disturbing the interaction of the ER protein Vesicle associated membrane protein (VAPB) and the mitochondrial protein tyrosine phosphatase interacting protein (PTPIP51) and it also reduces the uptake of calcium by mitochondria, which has detrimental effects on the Ca^2+^-dependent ATP synthesis pathway and the transportation of mitochondria in the neuron (Stoica et al., 2014). Notably, the loss of mitochondria-ER contact *via* the loss of VAPB-PTPIP51 contact, stimulates autophagy (Gomez-Suaga et al., 2017). It is known that reduced fusion and simultaneously increased mitochondrial fission can have damaging effects on the post-mitotic neurons. Of note, the overexpression of TDP-43 also promotes mitochondrial fragmentation with a concurrent increase in the levels of mitochondrial fission factors, dynamin related protein 1 (Drp1) and fission 1 (Fis1) (Xu et al., 2010). ALS patient-derived fibroblast cells carrying TDP-43 mutations have been reported to exhibit significantly increased Drp1 recruitment to the mitochondria and enhanced mitochondrial fragmentation. In fact, a selective peptide inhibitor of Fis1/Drp1 called P110 was found to greatly reduce this mitochondrial dysfunction thereby directly implicating the high levels of Drp1 in mitochondrial toxicity (Joshi et al., 2018) (Figure 7).

Cytoplasmic accumulation of TDP-43, which is a pathological feature of ALS, results in unsolicited interaction with various cellular organelles, primarily the mitochondria (Scotter et al., 2015). In 2012, full length and truncated TDP-43 proteins expressed in the motor neuron-like NSC-34 cells, were found to localize with mitochondria and cause its dysfunction (Hong et al., 2012) (Figure 7). Earlier in 2009, a proteomic study aimed to identify the interacting partners of TDP-43, revealed interactions with several mitochondrial ribosomal proteins (both the small and large subunit proteins) and several mitochondrial respiratory complex proteins including the alpha subunit-1 of mitochondrial F1 complex of ATP synthase (ATP5A1) (Freibaum et al., 2010). Recently, a proteomic screen has also revealed several TDP-43-interacting mitochondrial proteins including: mitochondrial porin, voltage-gated anion channel 1 (VDAC1), a crucial mitophagy receptor, prohibitin 2 (PHB2), mitochondrial fusion protein, mitofusin-2 (MFN2) and the mitochondrial respiratory complex proteins, ubiquinol-cytochrome creductase core protein I and II (UBQCRC1 and UBQCRC2) (Davis et al., 2018).

Inhibition of the TDP-43′s mitochondrial localization has been shown to rescue the TDP-43-mediated toxicity (Wang et al., 2016). Unlike the antioxidant enzyme SOD1-mediated ALS pathology, which is mostly attributed to the mitochondrial dysfunction, TDP-43 is believed to cause toxicity also by its RNA/DNA-binding regions (Bozzo et al., 2017). However, the observed presence of TDP-43 in the inner mitochondrial membrane fraction, and its preferential binding to the mitochondrial ND3 and ND6 mRNAs that encodes for the respiratory complex I subunits, have brought the focus back on the role of mitochondrial pathways in the TDP-43 toxicity (Wang et al., 2016). In fact, in a transgenic mouse model expressing the TDP-43 M337V mutant, inhibition of the mitochondrial localization could relieve the cognitive dysfunction and restore the mitochondrial function (Wang W. et al., 2017). This consolidates the interaction of TDP-43 with mitochondria as one of the crucial mechanisms in eliciting toxicity.

Mutations in the coiled helix domain containing 10 (CHCHD10) protein are linked to ALS, and the mutant CHCHD10 protein molecules are localized to the intermembrane space of mitochondria and are also found to interact with TDP-43 (Lehmer et al., 2018). CHCHD10 protein is involved in organizing of the cristae morphology and thereby playing a vital role in the mitochondrial integrity (Woo et al., 2017). Loss of function mutations in CHCHD10 are associated with the disassembly of mitochondrial contact site and cristae organizing system (MICOS) which has negative impact on the assembly of respiratory chain complex (Genin et al., 2016) (Figure 7). TDP-43 overexpression alters the CHCHD10 localization from the mitochondria to the nucleus and loss-of-function mutations in CHCHD10 induces cytoplasmic accumulation of TDP-43 (Woo et al., 2017). Interestingly, loss of mitochondrial integrity caused by mutations in CHCHD10 has been shown to be independent of the mitochondrial localization of TDP-43 (Genin et al., 2018).

Recently, when the A315T TDP-43 mutant was expressed in the mice model, though mitochondrial localization was detected there was no significant alteration in the mitochondrial bioenergetics, especially the oxidative phosphorylation (Kawamata et al., 2017). On the contrary, increased mitochondrial calcium uptake was observed, the potential implications of which need further investigation. As TDP-43 has been shown to bind to and stabilize the intermediates of the mitochondrial transcripts, including the electron transport chain transcripts, and as a considerable amount of TDP-43 is transported into the mitochondria even under normal conditions, furthermore studies are required to unearth the details of the molecular mechanisms of TDP-43 function and toxicity in relation to mitochondria (Izumikawa et al., 2017).

### Dysregulation of Metal Ion Homeostasis

The dysregulation of metal ion homeostasis has been implicated in a number of neurodegenerative diseases (Gaeta and Hider, 2005; Lovejoy and Guillemin, 2014; Chen P. et al., 2016). Increased metal ion levels can impart physiological insults like oxidative stress, mitochondrial dysfunction, protein misfolding, DNA damage, and ER stress etc. (Roos et al., 2006; Wright and Baccarelli, 2007; Dang et al., 2014). Strikingly, increased iron and iron-associated protein levels have been found in the ALS patients' brain cortex and blood sera (Veyrat-Durebex et al., 2014; Yu et al., 2014). Recently, in a TDP-43 mice model expressing the TDP-43 A315T mutant, a significant increase in the levels of zinc, manganese and copper ions were observed as compared to the control mice expressing the wild-type TDP-43 (Dang et al., 2014). The mechanism of the metal dysregulation caused by this mutant variant and the reason for the involvement of the spinal cord cells are unclear, however, the increment in the levels of these metal ions could be attributed to the oxidative stress and mitochondrial dysfunction, as observed by the elevated amounts of oxidized proteins in the spinal cord (Dang et al., 2014). In another study, zinc ions have also been shown to increase the TDP-43 aggregation propensity *in vitro* (Caragounis et al., 2010). On the contrary, certain copper-based complexes, such as Cu^II^(atsm) and Cu^II^(gtsm), have shown potential to significantly improve the phenotypes of the TDP-43- and SOD1-associated toxicity in the transgenic mice and the neuronal cell models (Parker et al., 2012; Roberts et al., 2014; Williams et al., 2016). Notably, the zinc ions could induce inclusion bodies formation and aggregation in the neuronal cell cultures, and this effect was not observed with copper or iron, indicating zinc-specific effects (Caragounis et al., 2010). In another study, a TDP-43 fragment with the RRM 1–2 domain *via* its histidine, cysteine, and glutamate residues that usually show affinity for zinc ions, was shown to aggregate in the presence of the zinc ions into ThT-staining rope-like aggregates (with hydrodynamic diameters: 300–1,000 nm) and also into small oligomeric structures (20–30 nm) (Garnier et al., 2017). Recently, Ash et al. demonstrated that heavy metals, such as lead, mercury and tin, can trigger aggregation and formation of nuclear inclusions of TDP-43 in the PC12 cell lines (Ash et al., 2018). The exposure to lead and methyl mercury was found to disrupt the TDP-43's homeostasis in the neuronal cells and dysregulate its splicing activity. Also, lead could decrease the TDP-43 solubility and promote the phase separation of TDP-43 *in vitro* in a dose-dependent manner (Ash et al., 2018). Thus, the relationship between metal ion content and the TDP-43 functions and aggregation need thorough investigation.

### Interference With Chromatin Remodeling

Notably, epigenetic processes, such as chromatin remodeling, histone modifications, and DNA methylation etc., are involved in several aspects of the neuronal function and development (Bastle and Maze, 2019). In fact, altered chromatin regulation might also be involved in the pathology of neurodegenerative diseases including the Alzheimer's, Huntington's and ALS diseases (Berson et al., 2018; Bastle and Maze, 2019). In an important study, TDP-43 was found to impair nucleosomal dynamics (Berson et al., 2017). Here, knockdown of the chromodomain helicase DNA binding protein 1 (CHD1), which is a nucleosome remodeling factor, in *Drosophila*, was shown to be associated with an increase in the number and size of stress granules, and also the percentage of cells exhibiting visible stress granules. TDP-43 was linked with impaired expression of heat shock response proteins, thereby decreasing survival, whereas the upregulation of CHD1 could restore their survival. Also, alteration of chromatin dynamics by TDP-43 due to abnormal histone clearance could be relieved upon CHD1 overexpression. In fact, co-immunoprecipitation showed that TDP-43 physically interacts with CHD1, and limits its recruitment to the chromatin machinery, possibly due to its aggregation-prone nature. Strikingly, the cytoplasmic accumulation of TDP-43 in ALS and FTLD brain cortex, was also found to be associated with decreased levels of its human ortholog CHD2, thereby indicating that aberrant chromatin remodeling might be involved in ALS (Berson et al., 2017) (Figure 6). Additionally, levels of the functional subunits of neuronal Brg1-associated factor (nBAF), which is another chromatin remodeling protein critical for the neuronal differentiation, dendritic extension and synaptic function, was reduced by mutant TDP-43 in the cultured motor neurons and in the ALS spinal motor neurons (Tibshirani et al., 2017). Also, the nuclear loss of TDP-43, or FUS, has been linked with concomitant nuclear retention of the mRNAs encoding the proteins involved in the formation of the nBAF complex. Furthermore, an ALS-linked TDP-43 M337V mutant, was shown to decrease the global expression levels of a key epigenetic marker, H3S10Ph-K14Ac in neuronal cells, whereas, the overexpression of wild-type TDP-43 caused an increase in the histone H3K9Me3 levels (Masala et al., 2018). In summary, TDP-43's role in epigenetics may have importance to the pathogenesis of ALS.

## TDP-43-Positive Inclusions in Other Neurodegenerative Diseases

Although, pathologically ubiquitinated and phosphorylated TDP-43 inclusions are commonly linked to neurodegeneration in ALS and FTLD-TDP patients (Arai et al., 2006; Neumann et al., 2006; Kwong et al., 2008), TDP-43-positive inclusion cross-immunoreactive pathology, has also been observed in the patients of several other neurological diseases such as: dementia with Lewy bodies (Higashi et al., 2007; Nakashima-Yasuda et al., 2007; Lin and Dickson, 2008), corticobasal degeneration (Uryu et al., 2008), progressive supranuclear palsy (PSP) (Yokota et al., 2010; Koga et al., 2017), dementia Parkinsonism ALS complex of guam (G-PDC) (Hasegawa et al., 2007; Geser et al., 2008), Pick's disease (Freeman et al., 2008; Lin and Dickson, 2008; Dardis et al., 2016), hippocampal sclerosis (Amador-Ortiz et al., 2007; Yokota et al., 2010), Alzheimer's disease, Huntington's disease and Parkinson's disease (Nakashima-Yasuda et al., 2007; Schwab et al., 2008; King et al., 2010b). Notably however, the localization of the TDP-43 aggregates in the brain varies among these different pathological conditions. While in the ALS, FTLD and G-PDC patients the TDP-43 inclusions are widespread in the brain, those in the Alzheimer's disease, Parkinson's disease and PSP patients are more prominent in the limbic region (Baloh, 2011).

Immunohistochemistry has revealed the presence of TDP-43 in the majority of the Huntington's disease patient cases, where it was found to be co-localized with the huntingtin protein in the dystrophic neurites and the intracellular inclusions but was absent from the intra-nuclear regions (Schwab et al., 2008). Schwab et al., using immuno-staining with phosphorylation-specific TDP-43 antibodies, have shown the presence of pathological and phosphorylated TDP-43 in the Huntington's disease samples (Schwab et al., 2008). In another study, TDP-43 was found to co-localize with the poly-glutamine aggregates in the cell culture models where a glutamine/asparagine-rich (Q/N) region from the C-terminal region of TDP-43, present between amino acids 320 and 367, was found to interact with the poly-glutamine aggregates (Fuentealba et al., 2010). Further studies from the nematode and cell culture models, have found that the poly-glutamine's toxicity can, in fact, be reduced by the suppression of the TDP-43 expression, proposedly due to a downstream effector protein, progranulin (PGRN) (Tauffenberger et al., 2013). The TDP-43 and PGRN-mediated effects on the huntingtin toxicity need further investigation using mammalian models and Huntington's disease patients (Tauffenberger et al., 2013).

Notably, immunoreactivity of TDP-43 has also been detected in the Alzheimer's disease brain, in about 75% of the patients (Amador-Ortiz et al., 2007; Higashi et al., 2007; Uryu et al., 2008; King et al., 2010b; Josephs et al., 2014). Immuno-histochemical analysis has found the presence of TDP-43 inclusions co-existing with the tau-positive neuro fibrillar tangles (NFTs) which suggests of its Aβ-42 independent role in the Alzheimer's disease cases (Higashi et al., 2007). However, *in vitro* studies have found that pre-formed TDP-43 aggregates, in fact, can prevent the maturation of the aggregating Aβ-42 into fibrils and rather arrest it into spherical oligomeric species (Fang et al., 2014). Notably, oligomeric Aβ-42 has already been implicated to be of high relevance to the neuro-toxicity in the Alzheimer's disease patients (Selkoe and Hardy, 2016). In another study, Herman et al., using mice Alzheimer's disease models, have found that the Aβ-42 amyloid can trigger the TDP-43 pathology, thus, the TDP-43 and Aβ-42 oligomers/aggregates appear to be capable of cross-seeding each other into toxic species (Herman et al., 2011; Fang et al., 2014; Chang et al., 2016).

Furthermore, TDP-43 proteinopathy has also been detected in the Parkinson's disease patients and also in the transgenic mice Parkinson's disease models, and the toxicity of α-synuclein to the dopaminergic neurons was found to be instigated by the concomitant over-expression of TDP-43 (Arai et al., 2009; Tian et al., 2011). Strikingly, TDP-43 has also been found to form cytoplasmic and sarcoplasmic inclusions in several other diseases such as: the Inclusion body myopathy with early-onset Paget disease and frontotemporal dementia (IBMPFD), sporadic IBM, myofibrillar myopathies, oculopharyngeal muscular dystrophy (OPMD) and distal myopathies with rimmed vacuoles (DMRV) (Weihl et al., 2008; Kusters et al., 2009; Olive et al., 2009; Salajegheh et al., 2009). In addition, TDP-43-positive inclusions have also been described in the skeletal muscles of the patients with sporadic inclusion body myositis (sIBM) and in IBM with mutations in the valosin-containing protein (VCP) (Weihl et al., 2008; Baloh, 2011). Considering the spectrum of diseases with TDP-43-positive inclusions, further investigation is required to illuminate whether the TDP-43 inclusions are indeed disease triggering, or rather merely an induced by-product effected by the other implicated primary aggregating proteins, such as Aβ-42 or α-synuclein etc.

## Therapeutic Strategies for ALS

### Targeting ALS-Related General Toxicity Mechanisms

Therapeutics of ALS is highly challenging as it is a complex disorder which involves numerous mechanisms linked with progressive motor neuron degeneration including the glutamate-mediated excitotoxicity, protein aggregation, increased oxidative stress, endoplasmic reticulum stress, mitochondrial dysfunction, neuro-inflammation and gene dysregulation etc. (Dugger and Dickson, 2017; Tan et al., 2017). In spite of the decades of extensive research, the sequence of events involved in the neuronal dysfunction in ALS remains largely unclear. Therapeutic options for ALS are very limited and thus far, no effective cure or diagnostic biomarkers have been developed for ALS (Mitsumoto et al., 2014; Petrov et al., 2017). Several efforts toward the development of therapeutics for ALS are in progress. These involve the identification of small molecules targeting the specific mechanisms causing the cellular dysfunction, some of which are discussed below.

#### Glutamate-Mediated Excitotoxicity

Glutamate is an important neurotransmitter in the mammalian nervous system. Excessive stimulation of glutamate receptors results in increased influx of Na^+^ and Ca^2+^ ions, which causes excitotoxicity, leading to neuronal injury or neuronal death (Heath and Shaw, 2002). A small molecule, riluzole, can inhibit glutamate excitotoxicity by regulating the release of glutamate, suppressing post-synaptic receptor activation and blocking voltage-sensitive sodium channels. In 1995, riluzole became the first FDA-approved drug for the treatment of ALS. However, it is only modestly effective in slowing the ALS disease progression, showing no effects on the disease symptoms and only improving the lifespan of ALS patients by 2–3 months (Bensimon et al., 1994; Miller et al., 2012). Also, a sodium channel blocker, mexiletine, which reduces the neuronal hyperexcitability and another glutamate antagonist, memantine, are presently under clinical trials for ALS treatment (De Carvalho et al., 2010; Weiss et al., 2016).

#### Oxidative Stress

Oxidative stress contributes to the motor neuron degeneration in ALS, and also affects the other cellular pathological mechanisms, such as the mitochondrial dysfunction and protein aggregation etc. (Barber et al., 2006). In 2017, a new anti-oxidant drug, edaravone (also called: radicava), became the first new FDA-approved drug for the treatment of ALS, in over two decades since riluzole. It is a free-radical scavenger and a potent anti-oxidant that alleviates the oxidative stress on the nerves and the vascular endothelial cells (Yoshino and Kimura, 2006; Takei et al., 2017).

#### Neuro-Inflammation

Evidence indicates that neuroinflammatory responses contribute to the progressive degeneration of neuronal cells in the ALS patients. An increase in the number of mast cells is associated with denervation of the neuromuscular junctions caused by degranulation of the mast cells, which release a mixture of serine proteases, histamine, and serotonin, etc. Masitinib is a selective tyrosine kinase inhibitor that mainly targets type III growth factor receptors like c-Kit, Lyn, and Fyn kinases, and is particularly effective in controlling the survival, differentiation, and degranulation of mast cells. Masitinib has been found to prevent the CNS neuro-inflammation by targeting the degranulation of the mast cells accumulating around the degenerating neuronal axons and by decreasing the release of inflammatory cytokines (Trias et al., 2016; Hammam et al., 2017). It can even target the microglia cells which are the resident macrophages of the brain. Masitinib has entered phase III trials for ALS therapeutics in 2017, as an add-on to riluzole (Mora and Hermine, 2017). Another small molecule immunomodulator, NP001, which reverses the pro-inflammatory response of the activated macrophages by producing intra-cellular chloramines, has also entered phase II trials for ALS treatment (Miller et al., 2014, 2015).

#### Muscle Troponin Activation

Muscular atrophy and decline of muscle strength, especially respiratory muscles, are among the key insults for ALS pathology. The functioning of muscle sarcomere depends upon the binding of myosin to actin which is regulated by the actin-associated proteins, tropomyosin, and troponin. The release of calcium ions from the sarcoplasmic reticulum and its binding to the troponin complex plays an important role in the fast skeletal muscle contraction. A small molecule, tirasemtiv can selectively sensitize the fast skeletal muscle sarcomere troponin to calcium ions, and slow down their release from the regulatory troponin complex, thereby amplifying the response of the muscles to neuromuscular inputs which improves the muscle function and muscle strength (Russell et al., 2012; Hansen et al., 2014; Hwee et al., 2014). Tirasemtiv had reached phase III trials for the ALS treatment however, the results were disappointing as the ALS patients reported poor tolerance. Recently, yet another next generation fast skeletal muscle activator, CK-2127107, has entered phase III trials and may potentially address the limitations of tirasemtiv (Andrews et al., 2017; Nace, 2017).

#### Heat-Shock Response Activation

Heat shock proteins, or chaperones, promote cell survival by refolding the misfolded proteins into their native functional conformations. The heat shock transcription factor 1 (HSF1) is a master regulator of the expression of several heat-shock proteins during stress conditions (Neef et al., 2011). A small molecule, arimoclomol, is a potent activator of HSF1 which also amplifies Hsp70 and Hsp90 expressions. In a recent study, arimoclomol showed HSF1-mediated reduction in the TDP-43 aggregate levels (Kieran et al., 2004; Kalmar et al., 2014; Wang P. et al., 2017). Arimoclomol has also shown promising results in the phase II trials for ALS.

#### Autophagy Induction

The cellular protein degradation machinery and autophagy pathways play a crucial role in clearing misfolded and aggregated proteins. The mammalian target of rapamycin (mTOR) kinase is an important protein involved in the regulation of cell signaling, protein synthesis, and autophagy pathway. Several small molecules, like trehalose and rapamycin, can induce protective autophagy and improve the neuronal health. Rapamycin, a small molecule inhibitor of mTOR, stimulates autophagy through the formation of autophagosomes from the phagophore and enhances protein degradation (Ravikumar et al., 2004; Bachar-Wikstrom et al., 2013). Rapamycin was shown to induce autophagy, improve memory and rescue motor dysfunction in a TDP-43 mouse model which manifested a decrease in the caspase-3 levels and the amount of cytoplasmic TDP-43 inclusions (Wang et al., 2012). Efficacy of rapamycin for the ALS treatment is being monitored in phase II clinical trials (Mandrioli et al., 2018).

### Targeting TDP-43's Aggregation and Clearance

#### Small Molecule Inhibitors of TDP-43 Aggregation

Small molecule interventions of the TDP-43 associated pathology need to aim at its aggregation behavior, stress granule dynamics, nucleo-cytoplasmic shuttling and caspase-resistance etc. Small molecule inhibitors of the amyloid-like aggregation, as well as stabilizers of the non-pathogenic native monomers or oligomers, may alleviate the neuronal toxicity. Tafamidis is the only, so far, FDA approved anti-amyloidogenic drug which is used for the treatment of transthyretin amyloidosis and it acts by arresting transthyretin into homo-tetrameric species (Bulawa et al., 2012). We have recently identified a TDP-43 aggregation inhibitor molecule which is an acridine-imidazole derivative (AIM4), using *in vitro* and yeast model (Prasad et al., 2016; Raju et al., 2016). In another study, using high-throughput screening, several compounds were identified that decrease the aggregation of TDP-43 into inclusion bodies and rescue the toxicity in the rat PC12 cells (Boyd et al., 2014). Furthermore, 4-aminoquinoline derivatives have been shown to alter the TDP-43's nucleic acid binding properties and enhance its caspase-mediated cleavage (Cassel et al., 2012). Also, inhibition of the TDP-43's accumulation into stress granules and inhibition of the C-terminal fragment aggregation, were reported in the ALS models treated with copper complexes Cu^II^(btsc) and Cu^II^(atsm), which proposedly act by slowly releasing the Cu^II^-ions within certain sub-cellular compartments like the stress granules (Donnelly et al., 2008; Crouch et al., 2009; Parker et al., 2012).

#### Heat Shock Proteins

In yeast, the over-expression of the chaperone Hsp104 has been vividly shown to efficiently dissolve certain yeast prion aggregates (Chernoff et al., 1995; Shorter and Lindquist, 2005; Liebman and Chernoff, 2012). Hsp104 has recently been used in the yeast models of several neurodegenerative disorders as a potential disaggregase (Jackrel and Shorter, 2014; Jackrel et al., 2014; Sweeny et al., 2015; Sweeny and Shorter, 2016; Torrente et al., 2016). Using random mutagenesis, engineered Hsp104 variants were obtained which showed capability of dissolving the aggregates of TDP-43, FUS, and α-synuclein (Jackrel et al., 2014). In fact, the mutants of Hsp104, A503V/S/C and V426L, could reduce the toxicity, suppress the aggregation and promote the nuclear localization of wild-type TDP-43 and an ALS-linked TDP-43 M337V mutant. Also, Hsp104 A503V and A503S variants, but not the wild-type Hsp104, displayed a propensity to dissolve the short TDP-43 filaments and amorphous structures *in vitro*, and similar observations were also documented for the FUS and α-synuclein fibrillar aggregates (Jackrel and Shorter, 2014; Jackrel et al., 2014). The cryo-EM structure of the hexameric Hsp104 is now available, which has revealed the mechanistic aspects of the substrate binding and disaggregation, and this may help in further optimization of its disaggregase activity (Gates et al., 2017). Following overexpression of Sis1, a yeast Hsp40 chaperone, reduction in the deleterious effects of the TDP-43 aggregation, was observed (Park et al., 2017). In fact, Sis1 could rescue the yeast cells from the TDP-43-associated toxicity, improve growth and survival, as well as protect from abnormal cellular morphologies, although there was no evidence for a direct physical association between Sis1 and TDP-43. Furthermore, overexpression of the mammalian Sis1 homolog, DNAJB1, in the primary rodent neurons could also relieve the TDP-43-mediated toxicity, suggesting that Sis1 and its related homolog might have neuroprotective effects for ALS (Park et al., 2017). Previously, heat shock has been shown to induce the reversible nuclear aggregation of TDP-43 (Udan-Johns et al., 2014). Interestingly, overexpression of DNAJB6, another Hsp40 protein, was found to suppress the formation of heat shock-induced TDP-43 nuclear aggregates. DNAJB6 was shown to be associated with the disordered C-terminal prion domain of TDP-43 and could possibly regulate not only its aggregation behavior but also its interaction with the other RNA binding partners (Udan-Johns et al., 2014).

#### Nuclear Import Receptors

Nuclear import receptors (NIRs), which are part of the nuclear pore machinery, have been shown to act as chaperones and disaggregases (Chook and Suel, 2011). In fact, karyopherin-β1 has shown an ability to decrease and reverse TDP-43 fibrillization possibly by associating with its classic nuclear localization signal (cNLS) sequence. Similarly, karyopherin-β2 could also decrease the fibrillization of some other RNA binding proteins—FUS, TATA-box binding protein associated factor 15 (TAF15), EWS RNA binding protein 1 (EWSR1), hnRNPA1, and hnRNPA2, by interacting with their proline-tyrosine nuclear localization signal (PY-NLS) sequences (Guo et al., 2018). Karyopherin-β2 was also found to manifest a propensity to dissolve the phase-separated droplets and aberrant fibril-containing hydrogels formed by FUS and hnRNPA1. Also, karyopherin-β2 could prevent the accumulation of these RNA-binding proteins into stress granules and restore their nuclear localization and cellular functions. Thus, condiserable interest exists in the nuclear importins as promising therapeutic targets (Hofweber et al., 2018; Yoshizawa et al., 2018).

#### Heat Shock Factors

Heat shock transcription factors have a significant role in maintaining the cellular proteostasis (Neef et al., 2011; San Gil et al., 2017). In an elegant study, heat shock factor1 (HSF1) was shown to reduce the levels of insoluble TDP-43 in the cell culture and mice ALS models (Chen H. J. et al., 2016). HSF1-mediated TDP-43 clearance was found to be closely associated with the chaperone Hsp70 and its co-chaperone DNAJB2a. Chen et al. suggest that DNAJB2a recognizes the insoluble TDP-43 aggregates and directs them toward Hsp70 for refolding and solubilization (Chen H. J. et al., 2016). Strikingly, activating HSF1 showed high transcriptional induction of Hsp40 and Hsp70 chaperones which significantly suppressed the TDP-43 aggregation into the inclusions bodies. Thus, harnessing the disaggregation potential of the HSPs and HSFs, as well as small molecule activators of HSF1 seem to be exciting prospects for TDP-43's anti-aggregation and therapeutics (Wang P. et al., 2017).

## Conclusions

The TDP-43 protein, by virtue of its versatile functions in RNA metabolism and homeostasis, has emerged as a vital protein for cellular health. Supporting its importance, aberrations in the TDP-43 homeostasis due to imbalance in its nucleocytoplasmic distribution, genetic mutations, aberrant post-translational modifications or aggregation, is increasingly being accepted as a causative of mis-regulation of RNA homeostasis and cytotoxicity. A few facets of the TDP-43-induced toxicity, such as mitotoxicity and proteosomal overload etc. have been unearthed, but how and in which sequence the toxicity mechanisms ensue leading to the neurodegeneration, remain poorly understood. The presence of TDP-43-positive inclusions in several other neurodegenerative diseases, in addition to ALS and FTLD, suggest of a more wide-spread and vital role of TDP-43 in the general process of neuro-degeneration. Thus, targeting the TDP-43 dyshomeostasis, may hold the key to finding common therapeutics, applicable to a multitude of neurodegenerative diseases.

## Author Contributions

BP directed the manuscript preparation. AP, VB, VS, AG, and BP wrote the manuscript.

### Conflict of Interest Statement

The authors declare that the research was conducted in the absence of any commercial or financial relationships that could be construed as a potential conflict of interest.
